# Dissecting dynamics and differences of selective pressures in the evolution of human pigmentation

**DOI:** 10.1242/bio.056523

**Published:** 2021-02-09

**Authors:** Xin Huang, Sijia Wang, Li Jin, Yungang He

**Affiliations:** 1 Chinese Academy of Sciences Key Laboratory of Computational Biology, Chinese Academy of Sciences-Max Planck Society Partner Institute for Computational Biology, Shanghai Institute of Nutrition and Health, Shanghai Institutes for Biological Sciences, University of Chinese Academy of Sciences, Chinese Academy of Sciences, Shanghai 200031, China; 2 State Key Laboratory of Genetic Engineering and Ministry of Education Key Laboratory of Contemporary Anthropology, Collaborative Innovation Center for Genetics and Development, School of Life Sciences, Fudan University, Shanghai 200433, China; 3 Key Laboratory of Medical Epigenetics and Metabolism, Institutes of Biomedical Sciences, Fudan University, Shanghai 200032, China

**Keywords:** Population genetics, Natural selection, Human evolution, Human pigmentation, Complex traits

## Abstract

Human pigmentation is a highly diverse and complex trait among populations and has drawn particular attention from both academic and non-academic investigators for thousands of years. Previous studies detected selection signals in several human pigmentation genes, but few studies have integrated contribution from multiple genes to the evolution of human pigmentation. Moreover, none has quantified selective pressures on human pigmentation over epochs and between populations. Here, we dissect dynamics and differences of selective pressures during different periods and between distinct populations with new approaches. We use genotype data of 19 genes associated with human pigmentation from 17 publicly available datasets and obtain data for 2346 individuals of six representative population groups from across the world. Our results quantify the strength of natural selection on light pigmentation not only in modern Europeans (0.0259/generation) but also in proto-Eurasians (0.00650/generation). Our results also suggest that several derived alleles associated with human dark pigmentation may be under positive directional selection in some African populations. Our study provides the first attempt to quantitatively investigate the dynamics of selective pressures during different time periods in the evolution of human pigmentation.

This article has an associated First Person interview with the first author of the article.

## Abstract (precis)

**Summary:** This study developed a novel approach to estimate selection strength over time and applied it on the evolution of human pigmentation.

## INTRODUCTION

Human pigmentation – the colour of human skin, hair, and eye – is one of the most diverse traits among populations. Its obvious diversity has attracted attention from both academic and non-academic investigators for thousands of years, as noted by Charles Darwin one century ago (Darwin, 1889) and as noticed by ancient Egyptians more than 4000 years ago (Norton, 2005). Why human pigmentation diverges, however, remains a central puzzle in human biology (Rees and Harding, 2012). Some researchers have proposed that the diversity of human pigmentation is adapted for the global difference in ultraviolet radiation (UVR) and driven by natural selection (Jablonski and Chaplin, 2000; Barsh, 2003; Parra, 2007; Jablonski and Chaplin, 2010). Dark skin may prevent sunburn amongst individuals in low latitude areas with high UVR, while light skin may protect infants against rickets in high latitude areas with low UVR (Jablonski and Chaplin, 2000; Parra, 2007; Chaplin and Jablonski, 2009; Jablonski and Chaplin, 2014, 2017; Cuthill, et al., 2017; Hochberg and Hochberg, 2019; Wolf and Kenney, 2019). Human pigmentation, especially skin pigmentation, is one of the traits that are under strong natural selection during the human dispersal out of Africa, because it is the first barrier between human body and living environment. A better understanding of how natural selection shapes the diversity of human pigmentation could provide relevant and beneficial information for public health (Jablonski and Chaplin, 2000; Barsh, 2003; Parra, 2007).

During the last decade, many studies have applied statistical tests to detect signals of natural selection in several human pigmentation genes (Izagirre et al., 2006; Voight et al., 2007; Lao et al., 2007; Myles et al., 2007; Norton et al., 2007; Pickrell et al., 2009; Beleza et al., 2013; Hider et al., 2013). These genes encode different proteins, including: signal regulators – ASIP, KITLG, MC1R – stimulating the melanogenic pathway; possible enhancers – BNC2, HERC2 – regulating pigmentation gene expression; important enzymes – TYR, TYRP1, DCT – converting tyrosine into melanin; putative exchangers – OCA2, SLC24A4, SLC24A5, SLC45A2, TPCN2 – controlling the environment within melanosomes; and an exocyst complex unit and molecular motor – EXOC2, MYO5A – conveying vesicles and organelles within the cytoplasm (Abdel-Malek et al., 2001; Rebbeck et al., 2002; Duffy et al., 2004, 2007; Graf et al., 2005; Sulem et al., 2007, 2008; Anno et al., 2008; Han et al., 2008; Ito and Wakamatsu, 2008; Kayser et al., 2008; Sturm and Duffy, 2012; Visser et al., 2012, 2014; Guenther et al., 2014). These proteins work at different stages of the melanogenic pathway, illustrating that human pigmentation is a complex trait affected by multiple genes with different roles.

Previous studies applied two groups of methods to detect natural selection. One group of methods detects unusually long extended haplotype homozygosity (Izagirre et al., 2006; McEvoy et al., 2006; Voight et al., 2007; Lao et al., 2007; Myles et al., 2007; Norton et al., 2007; Pickrell et al., 2009; Donnelly et al., 2012; Beleza et al., 2013). The other group of methods identifies extremely local population differentiation (Izagirre et al., 2006; Lao et al., 2007; Myles et al., 2007; Norton et al., 2007; Pickrell et al., 2009; Hider et al., 2013). By applying both groups of methods, previous studies have aimed to interpret the evolution of individual pigmentation genes; however, few studies have integrated contributions from multiple genes to the evolution of human pigmentation. Moreover, none of these studies have quantitatively investigated the historical selective pressures of pigmentation genes during different epochs and compared the differences of selective pressures between distinct populations. Therefore, it is necessary to perform an extensive quantification of selective pressures on human pigmentation using a creative approach.

## RESULTS

### The model

On the basis of a previous study (He et al., 2015), we measure selective pressures by (genic) selection coefficients. For any single nucleotide polymorphism (SNP) *L*, we can estimate the expectation of the selection (coefficient) difference per generation between populations *i* and *j* by(1)
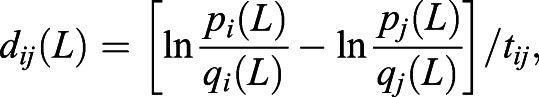
where *p* and *q* are the frequencies of derived and ancestral alleles in a population, respectively; and *t_ij_* is the divergence time of the populations *i* and *j* from their most recent common ancestor. Details of the calculations are described elsewhere (He et al., 2015).

We can extend Eqn 1 to summarise selection differences of multiple SNPs by assuming additive fitness and weak linkage disequilibrium. In general, for a trait with *n* SNPs, the expectation and variance of the total selection difference in this trait is(2)
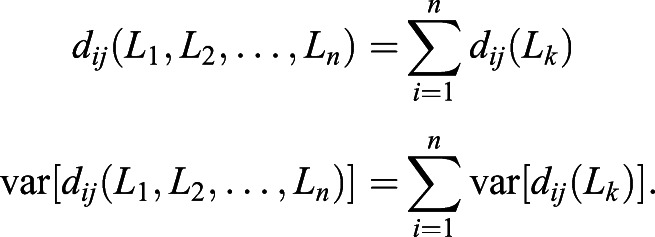


Here, we take two bi-allelic SNPs as an example. Using Eqn 1, we can estimate the selection difference of a trait affected by two SNPs between populations *i* and *j* using
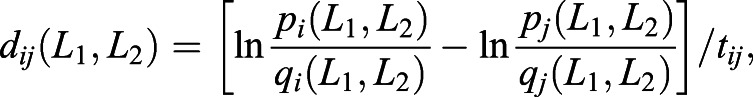


where *p*(*L*_1_, *L*_2_) is the frequency of the combination carrying two alleles associated with one possible outcome of a trait, such as light pigmentation; *q*(*L*_1_, *L*_2_) is the frequency of the combination carrying two alleles associated with another outcome of the same trait, such as dark pigmentation. With linkage equilibrium between *L*_1_ and *L*_2_, we have *p*(*L*_1_, *L*_2_)=*p*(*L*_1_)*p*(*L*_2_) and *q*(*L*_1_, *L*_2_)=*q*(*L*_1_)*q*(*L*_2_). Thus
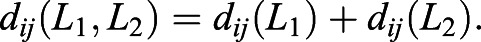
We further assume the distribution of selection difference in each SNP is independent. Therefore, the variance of *d*_*ij*_(*L*_1_, *L*_2_) is
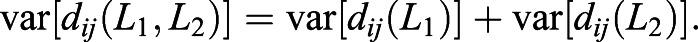
The confidence interval (CI) of *d*_*ij*_(*L*_1_, *L*_2_) becomes 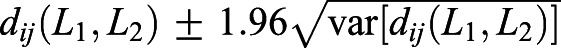.

**Fig. 1.**
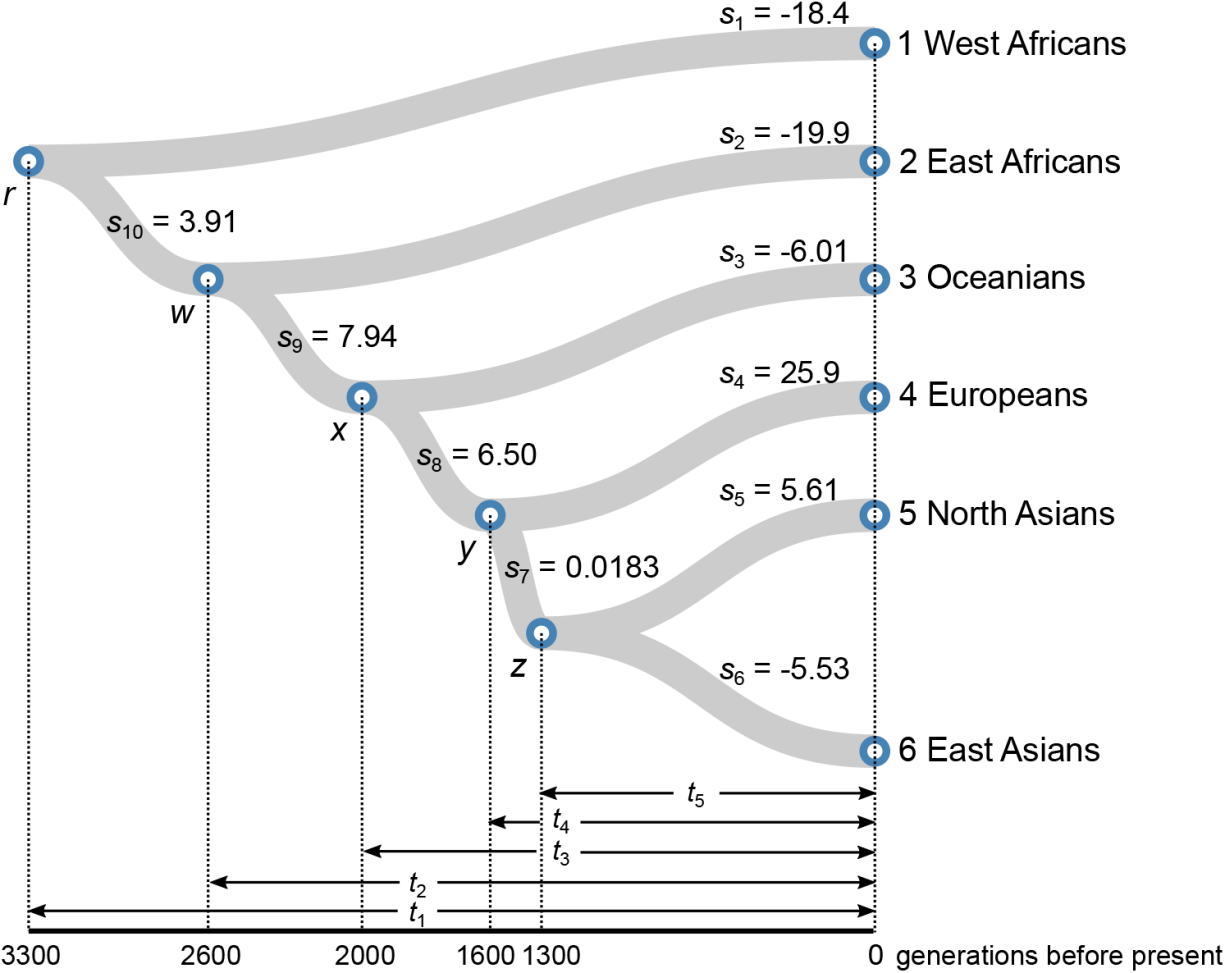
**Time-varied selective pressures on an evolutionary tree.** We model the evolutionary history of six representative human populations as a binary tree. Here, *s_i_* (*i*=1, 2, …, 10) denotes the selection coefficient of the *i*-th epoch and can be obtained by estimating selection differences between paired populations. Divergence times between populations are based on previous studies (Schaffner et al., 2005; Oppenheimer, 2012; Schiffel and Durbin, 2014; Mondal et al., 2016). We assume one generation time is ∼30 years. We obtain the optimal solution deviated least from neutral evolution using a probabilistic approach. The numbers (×10^−3^/generation) on the branches are the optimal solution. In the solution, numbers larger than zero suggest natural selection favoured light pigmentation, while those less than zero indicate natural selection preferred dark pigmentation.

Based on Eqn 2, we develop a new approach for dissecting historical selective pressures over epochs of the human evolutionary history. We simplify the evolutionary history of six representative human populations as a binary tree (Fig. 1). We can further divide the selection difference between paired populations into multiple terms if there are multiple branches between them. We further assume the selective pressure on each branch is constant over time. Let *k* denote the most recent common ancestor of *i* and *j*, we can divide *d_ij_* in Eqn 1 into separate terms:
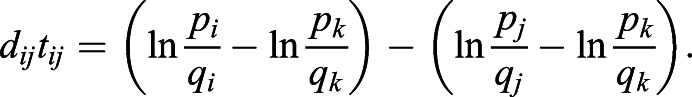
For example, using the notations and demographic model in Fig. 1, we can estimate the total selection difference between East and West Africans as
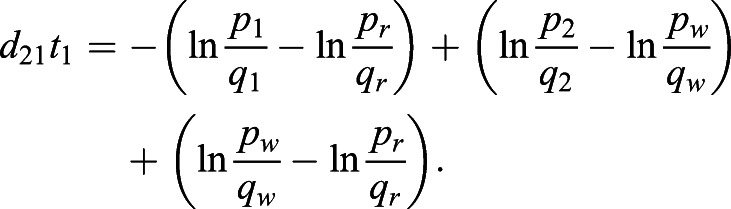
Let 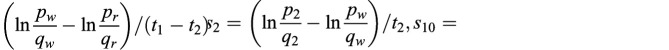, then we have
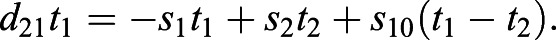
Therefore, we can represent the selection difference between paired populations as a combination of selection coefficients during different time periods. Using matrix algebra and the notations in Fig. 1, we can obtain Eqn 3, where
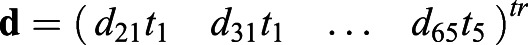

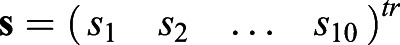

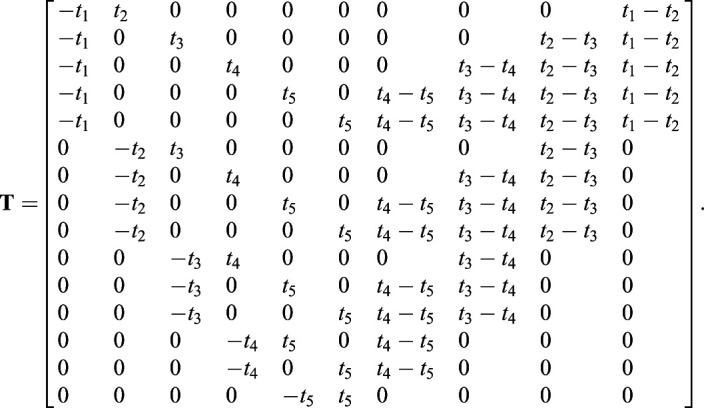

Using matrix algebra, we can represent the selection differences of all the paired populations in Fig. 1 as(3)
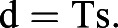


To obtain the optimal solution, we propose a probabilistic approach following the principle of minimum evolution (Cavalli-Sforza and Edwards, 1967; Edwards, 1996). Under neutral evolution (NE), we consider each estimated *s* as an independent random variable following a normal distribution with a mean of zero and a variance of *σ*^2^. For each solution with ten variables, the summation below follows a chi-square distribution with ten degrees of freedom:
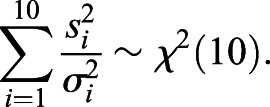
Therefore, we have Pr(|**s**|^2^>|**s***_a_*|^2^|NE)≥Pr(|**s**|^2^>|**s***_b_*|^2^|NE), if |**s***_a_*|^2^≤|**s***_b_*|^2^ for solutions *a* and *b*. Here, |**s**|^2^ is the norm of a vector **s**. In other words, we can choose the most conservative solution with the least deviation from neutral evolution using a probabilistic approach. Thus, the optimal solution s* is the minimum norm solution of Eqn 3:(4)
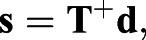
where **T**^+^ is the Moore-Penrose inverse of **T**.

### Selective pressures over epochs

We applied our new approach with genotype data of worldwide populations from 17 publicly available datasets (Table S1). After data preparation (Materials and Methods), we obtained 2346 individuals and grouped them into six population groups based on their geographic locations (Table S2). We also selected 52 SNPs in 19 genes for analysis due to their association with human pigmentation in published genome-wide association studies (GWAS) or phenotype prediction models (Table S3; Rebbeck et al., 2002; Bonilla et al., 2005; Graf et al., 2005; Lamason et al., 2005; Stokowki et al., 2007; Miller et al., 2007; Anno et al., 2008; Han et al., 2008; Kayser et al., 2008; Sturm et al., 2008; Sulem et al., 2008; Eiberg et al., 2008; Branicki et al., 2009; Edwards et al., 2010; Branicki et al., 2011; Donnelly et al., 2012; Visser et al., 2012; Hart et al., 2013; Jacobs et al., 2013; Praetorius et al., 2013; Walsh et al., 2013; Guenther et al., 2014; Murray et al., 2015; Yang et al., 2016; Ainger et al., 2017; Crawfold et al., 2017). We then used Eqn 2 with 30 SNPs not in strong linkage disequilibrium (*r*^2^<0.8) to estimate the total selection differences on human pigmentation (Materials and Methods). The maximum differences were observed between Europeans and the two African populations, while the minimum difference was observed between West and East Africans (Table 1). The estimated 95% confidence intervals (CI) indicate we cannot rule out the possibility that genetic drift caused the difference between East and West Africans, as well as between Oceanians and East Asians (Table 1). We further assessed the significance levels of the observed selection differences by randomly sampling 10,000 sets of 30 SNPs in the genome and obtained the empirical distributions of population differences (Fig. S1). The differences from random sets of SNPs are close to zero, which is consistent with a recent study (Fortier et al., 2019, preprint) that suggests no genome-wide difference in the strength of natural selection between human populations. Whereas those from SNPs associated with human pigmentation are significantly departure from zero (Fig. S1), indicating that most population differences on SNPs associated with human pigmentation are possibly contributed by natural selection.

**Table 1.**
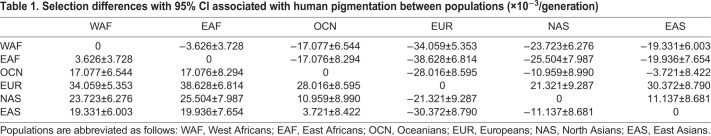
**Selection differences with 95% CI associated with human pigmentation between populations (×10^−3^/generation)**

We then solved the linear system (Eq. 4) with the observed selection differences on human pigmentation (Table 1). Our estimate shows that the modern European lineage had the largest selective pressure (*s*_4_=0.0259/generation) on light pigmentation than the other branches (Fig. 1), suggesting that recent natural selection favoured light pigmentation in Europeans. Recent studies using ancient DNA could support our observation of recent directional selection in Europeans (Wilde et al., 2014; Mathieson et al., 2015). Our results also reveal the selective pressure on light pigmentation in the ancestral population of Europeans and East Asians (*s*_8_=0.00650/generation). This shared selection is also supported by other studies, revealing that *ASIP*, *BNC2*, and *KITLG* were under directional selection before the divergence of ancestral Europeans and East Asians (Donnelly et al., 2012; Beleza et al., 2013). We further applied SLiM 2 to examine whether the optimal solution could reproduce the observed selection differences (Haller and Messer, 2017) (Table 1). We set up a human demographic model according to previous studies and used the optimal solution as selection coefficients during different periods (Materials and Methods). The simulated selection differences are close to the data and little affected by the initial frequency of the beneficial allele (Fig. S2). This also illustrates that though we assume genic selection, our model could approximate genotypic selection in diploids (Materials and Methods).

### Selection differences between populations

We also separately quantified selection differences of individual SNPs associated with human pigmentation (Table S4) using Eq. 1. Ten SNPs were removed because of their low derived allele frequencies among populations in our data (Materials and Methods). Statistical tests suggest that selective pressures in many loci differed significantly between populations (*P*<0.05). The remaining 42 SNPs were categorised into five groups (Fig. 2).

**Fig.**
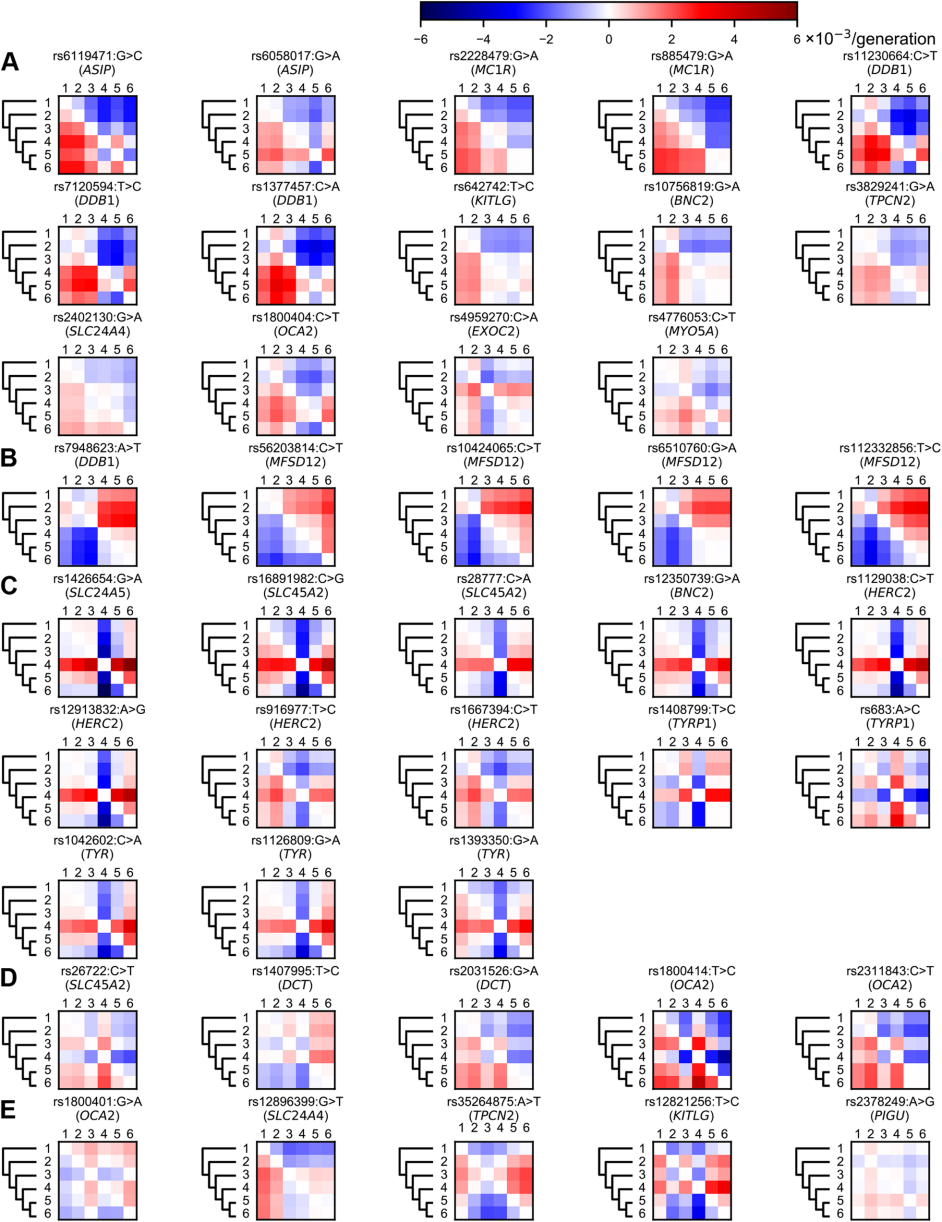
**2. Selection differences in individual loci between populations.** We used Eqn 1 to quantify the selection differences of 42 SNPs associated with human pigmentation, and categorised them into five kinds of patterns: (A) derived alleles affected by Eurasian-shared selection; (B) derived alleles affected by African-specific selection; (C) alleles affected by European-specific selection; (D) alleles affected by Asian-specific selection; and (E) others. Red colour indicates selective pressures of populations in rows are larger than those in columns; blue colour indicates selective pressures of populations in rows are smaller than those in columns. Populations are abbreviated as follows: 1, West Africans (*n*=493); 2, East Africans (*n*=59); 3, Oceanians (*n*=32); 4, Europeans (*n*=701); 5, North Asians (*n*=114); 6, East Asians (*n*=947).

In the first group, derived alleles may be affected by Eurasian-shared selection (Fig. 2A). Among these SNPs, rs6119471 (*ASIP*) has large selection differences between Eurasians and Africans (Table S4). The derived allele of rs6119471 (*ASIP*) is almost fixed across Eurasians but maintains a low frequency in Africans (Fig. S4). Recent studies applied this SNP to predict dark/non-dark pigmentation phenotype in human (Spichenok et al., 2011). This may be explained by different selective pressures on this SNP among populations. Our results also indicate that two SNPs in *MC1R* (rs2228479 and rs885479) largely differ between Eurasians and Africans (Table S4). Previous studies used variants in *MC1R* to solve a long-standing puzzle, regarding whether light pigmentation in low UVR areas is caused by directional selection or the relaxation of selective pressures (Rana et al., 1999; Harding et al., 2000; Wilde et al., 2014). The relaxation of selective pressures would suggest that the diversity of *MC1R* variants increased in Eurasians due to the lack of selective constraints. In this scenario, the genetic diversity of *MC1R* variants could be largely attributed to genetic drift. In contrast, directional selection would suggest that *MC1R* variants were under positive selection in Eurasians. In this scenario, genetic drift cannot explain the genetic divergence of *MC1R* between Africans and Eurasians. Our statistical results show that the divergences of rs2228479 and rs885479 between Eurasians and Africans are highly significant departure from neutral evolution (Table S4), suggesting that directional selection is the more likely explanation. Experimental evidence suggests that the derived allele of rs2228479 could cause lower affinity for alpha-melanocyte stimulating hormone than the ancestral allele (Xu et al., 1996). Another study showed that the derived allele of rs885479 carries a lower risk of developing freckles and severe solar lentigines than the ancestral allele in East Asians (Motokawa et al., 2007). These studies revealed the potential roles of these *MC1R* variants in pigmentation phenotypes.

In the second group, derived alleles may be affected by African-specific selection (Fig. 2B). All these derived alleles are in/near two genes (*DDB1* and *MFSD12*) and were recently associated with human dark pigmentation (Crawfold et al., 2017). The previous study (Crawfold et al., 2017) did not find signals of positive selection at *MFSD12* using Tajima's D or iHS. Our method (He et al., 2015; Huang et al., 2019) shows that these SNPs in *MFSD12* differ significantly between Africans and Eurasians, possible signals of directional selection (Table S4). From the first and second groups, we can observe that directional selection not only affects derived alleles associated with light pigmentation in Eurasians, but also influences derived alleles associated with dark pigmentation in Africans. This observation suggests that human pigmentation is under directional selection with diversifying orientations among different populations. Thus, the previous view that the dark pigmentation in Africans is the result of purifying selection on ancestral alleles is incomplete.

The third and fourth groups display European- and Asian-specific selection, respectively (Fig. 2C and D). One notable SNP is rs1426654 (*SLC24A5*), which had the largest selection difference between Europeans and East Asians in our study (0.005774/generation). Previous studies reported that this SNP is under strong directional selection in Europeans (Izagirre et al., 2006; Voight et al., 2007; Lao et al., 2007; Myles et al., 2007; Norton et al., 2007). Another notable SNP is rs1800414 (*OCA2*), which has large selection differences between East Asians and other populations. This reveals a potential role of rs1800414 (*OCA2*) on light pigmentation in East Asians. Several studies have suggested directional selection on this SNP in East Asians (Edwards et al., 2010; Yang et al., 2016). These large selection differences indicate the significant contributions of these SNPs to light pigmentation in Europeans and East Asians, respectively. Other SNPs in these groups also support the hypothesis that recent natural selection for light pigmentation independently occurred in Europeans and Asians since they diverged (Norton et al., 2007; Edwards et al., 2010; Yang et al., 2016). Interestingly, Oceanians comprise both African-specific (*DDB1*) and Asian-specific (*OCA2*) selection. However, due to limited sample size of Oceanians in our data from publicly available resources (Table S2), it should be cautious to interpret these results. It would be helpful to analyse larger datasets of Oceanians to confirm our observation.

The last group includes the five remaining SNPs (Fig. 2E), which exhibit specific selection differences between limited population pairs. Among them, the derived allele of rs1800401 (*OCA2*) and the ancestral allele of rs12896399 (*SLC24A4*) are both associated with dark pigmentation (Table S2). Only rs12896399 (*SLC24A4*) differs significantly between West Africans and Eurasians (Table S4). This may be a selection signal associated with dark pigmentation in West Africans, again indicating possible genetic diversity within African populations. We note that rs35264875 (*TPCN2*) and rs12821256 (*KITLG*) might be affected by selection in both East Africans and Europeans. A recent study showed that rs12821256 might have large effect on the skin pigmentation in South Africans (Martin et al., 2017). The other two SNPs, rs3829241 (*TPCN2*) and rs642742 (*KITLG*), also differ between Eurasians and Africans (Fig. 2A). These similar patterns of *TPCN2* and *KITLG* might suggest some connection between them.

## DISCUSSION

Compared with previous studies (Izagirre et al., 2006; Voight et al., 2007; Lao et al., 2007; Myles et al., 2007; Norton et al., 2007; Pickrell et al., 2009; Beleza et al., 2013; Hider et al., 2013; Wilde et al., 2014), our study has three advantages. First, our approach considers the fluctuation of selective pressures over epochs, an important factor in evolution (Crow and Kimura, 2009) that was ignored by previous studies. Our results provide more information about the dynamics of selective pressures during human evolution. Second, we summarise selective pressures based on multiple human pigmentation genes (Eq. 2), while previous studies usually tested selection signals in individual human pigmentation genes. Moreover, we simultaneously interpret selective pressures in multiple populations, whereas previous studies separately investigated selection signals in single population. Third, we do not need to assume population continuity as in those ancient DNA studies (Wilde et al., 2014; Mathieson et al., 2015), because our study is based on genetic data from only present-day populations.

We note that our investigation has several limitations. First, our model is based on the infinite population size model. The limited sample size would affect our results, therefore, we grouped populations into large population groups based on their geographic locations to mitigate the effect of sample size. Analysis of data with larger sample size could improve our estimate, as more and more genomic datasets become available. Second, although we chose the solution that deviates least from neutral evolution as the optimal solution, we cannot exclude the possibility of other solutions. This reflects the difficulty of analysing historical selective pressures, which is a well-recognised challenge in population genetics (Crow and Kimura, 2009). Our solution provides a first step toward resolving the dynamics of selection in the evolution of human pigmentation. This solution may be improved by combining both ancient and modern human genetic data, as well as by using a Bayesian framework for inference. Adding more population groups would also possibly improve the solution, because this would provide more constraints in the linear system (Eq. 4). Third, our results may be affected by a severe bottleneck. A recent study (Terhorst et al., 2017) suggests a more severe Out-of-Africa bottleneck in human evolutionary history than in the model used in our simulation. This would probably reduce the selection differences between Eurasians and Africans, leading to an underestimation of selective pressures. Fourth, our results may also be affected by population migration and sub-structure. We used knowledge from previous studies, principle component analysis and *F*_3_ test to rigorously prune potential admixed populations, including South Asians, Central Asians, the Middle East People and Americans. Removing these populations would lose information of selective pressures on human pigmentation in these lineages; however, as a first step to explore the historical selective pressures in the evolution of human pigmentation, we focused more on reducing the bias induced by population admixture. New methods explicitly accounting for population admixture would be helpful to provide more comprehensive view on the dynamics of selective pressures during the evolution of human pigmentation. Besides, we demonstrate that our estimate provides lower bounds of selection differences on human pigmentation when migration or sub-structure exists (Materials and Methods). Fourth, the human pigmentation SNPs used in our study may be biased. For example, our results indicate small genetic differences on human pigmentation between Oceanians and East Asians (Table 1), while recent studies (Martin et al., 2017) suggest Oceanians are darker than East Asians in skin pigmentation using melanin index. One possible reason is that some Oceanian-specific or East-Asian-specific SNPs are missing. This is because we selected candidates based on results from published GWAS or phenotype prediction models, and most of these studies used samples with European ancestry (Sirugo et al., 2019). More studies on non-European populations could resolve this missing diversity and enhance our knowledge on the evolution of human pigmentation. Finally, we noticed that our model is a simple model. Other biological factors, such as linkage disequilibrium between SNPs, sexual selection, and different levels of vitamin D among human populations, may be possible to be integrated into a more comprehensive model based on this simple model.

To summarise, we extended an established method (He et al., 2015) to dissect dynamics of selective pressures over epochs. Our study provides the first attempt to resolve time-varied selective pressures in the evolution of human pigmentation. Our study also provides information on differences of selective pressures between distinct population groups. Further studies are in progress to verify our present views on the evolution of human pigmentation.

## MATERIALS AND METHODS

### Data preparation

Seventeen datasets (Li et al., 2008; Teo et al., 2009; Behar et al., 2010; Rasmussen et al., 2010; The, 1000 Genomes Project Consortium, 2010; The International HapMap 3 Consortium, 2010; Metspalu et al., 2011; Pagani et al., 2012; Yunusbayev et al., 2012; Di Cristofaro et al., 2013; Fedorova et al., 2013; Xing et al., 2013; Kovacevic et al., 2014; Raghavan et al., 2014; Yunusbayev et al., 2015; Mondal et al., 2016; Pagani et al., 2016) containing genotype data from worldwide human populations were obtained from the listed resources (Table S1). After downloading, all the genotype data were liftovered to genomic coordinates using the Human Reference Genome Hg19. A merged dataset containing 6531 individuals was obtained after removing duplicated and related individuals. After merging, SNPs with call rate less than 0.99 or individuals with call rate less than 0.95 were removed. SNPs in strong linkage disequilibrium were further removed by applying a window of 200 SNPs advanced by 25 SNPs and an *r*^2^ threshold of 0.8 (--indep-pairwise 200 25 0.8) in PLINK 1.7 (Purcell et al., 2007). This LD-pruning was applied to each population separately. The remaining 61,597 SNPs were used for further analysis. In order to mitigate the bias induced by population migration, potential admixed populations, such as the Middle East People and South Asians, were excluded according to previous studies (Li et al., 2008; Teo et al., 2009; Behar et al., 2010; Rasmussen et al., 2010; The 1000 Genomes Project Consortium, 2010; The International HapMap 3 Consortium, 2010; Metspalu et al., 2011; Pagani et al., 2012; Yunusbayev et al., 2012; Di Cristofaro et al., 2013; Fedorova et al., 2013; Xing et al., 2013; Kovacevic et al., 2014; Raghavan et al., 2014; Yunusbayev et al., 2015; Mondal et al., 2016; Pagani et al., 2016), principal component analysis (PCA) using SMARTPCA (version: 13050) from EIGENSOFT (version: 6.0.1) (Patterson et al., 2006; Price et al., 2006), and *F*_3_ test using ADMIXTOOLS (version: 3.0) (Patterson et al., 2012). Finally, 2346 individuals were obtained and divided into six groups according to their geographic regions for further analysis. These groups are West Africans, East Africans, Oceanians, Europeans, North Asians and East Asians. The PCA plot (Fig. S3) shows that these 2346 individuals were properly separated into six population groups.

### Data imputation

Genotypes of 19 human pigmentation genes with 500-kb flanking sequences on both sides were obtained from the genotype datasets. Haplotype inference and genotype imputation were performed on the selected genotypes using BEAGLE 4.1 (Browning and Browning, 2007, 2016) with 1000 Genomes phase 3 haplotypes as the reference panel. During phasing and imputation, the effective population size was assumed to be 10,000 (*N_e_*=10,000), and the other parameters were set to the default values. Ten SNPs (rs1110400, rs11547464, rs12203592, rs1800407, rs1805005, rs1805006, rs1805007, rs1805008, rs1805009, rs74653330) were removed because of their low derived allele frequencies in our datasets after imputation (Fig. S4). Because rs12203592 (*IRF4*) was removed, 18 genes with the remaining 42 SNPs were used for further analysis.

### Estimating selection differences between populations and selective pressures over epochs

We used Eqn 1 to estimate the selection differences of the remaining 42 SNPs. We then used Eqn 2 and selected 30 SNPs not in strong linkage disequilibrium (*r*^2^<0.8) as well as known phenotypes to estimate the total selection differences on human pigmentation between populations. These SNPs were rs3829241, rs56203814, rs916977, rs1800414, rs10424065, rs6119471, rs1408799, rs11230664, rs4959270, rs1800401, rs2378249, rs1042602, rs12350739, rs6058017, rs12821256, rs1393350, rs1426654, rs642742, rs6510760, rs1129038, rs2228479, rs35264875, rs12896399, rs26722, rs16891982, rs885479, rs28777, rs1800404, rs10756819, rs2402130. To dissect selective pressures over epochs, we applied Eqn 4 with the total selection differences from the selected 30 SNPs and the divergence times shown in Fig. 1.

### Reproducing the observed selection differences from the optimal solution

We used SLiM 2 (version: 2.6) (Haller and Messer, 2017) to simulate a demographic model of human evolution (Fig. S5) to examine whether the optimal solution could reproduce the observed selection differences. We varied the initial frequency of the beneficial allele with 0.001 and 0.01. We divided the optimal solution by 30 to obtain the average selection coefficient for each SNP, because we used 30 SNPs to estimate the total selection differences on human pigmentation. We used the effective population size of each population estimated by previous studies (McEvoy et al., 2011; Mezzavilla and Ghirotto, 2015). We set both the mutation rate and the recombination rate to 1×10^-^^8^ per generation per site. In each run, we simulated a fragment with 10^6^ base pairs, and set the 50,000th site under selection. We repeated each set of parameters more than 10,000 times and analysed those results in which beneficial alleles were not fixed or lost in all the populations. We compared the average selection differences from simulation with the observed selection differences. We noticed that the selection coefficient in SLiM 2 measures differences in fitness between genotypes instead of alleles. We can transform the selection coefficient of genotypes into that of alleles as follows. Let the fitness of the ancestral allele *A* be 1, and the relative fitness of the derived allele *a* is *e^s^*. When *s* is close to 0, we can approximate *e^s^* as 1+*s* using the Taylor series. The fitness of genotype *aa* becomes (1+*s*)^2^=1+2*s*+*s*^2^≈1+2*s*, and the fitness of genotype *Aa* is 1+*s*=1+0.5*s*′. If *s′* is the selection coefficient in SLiM 2, then *s*′=2 s; and the dominance coefficient becomes 0.5. Simulations were performed in Digital Ocean (https://cloud.digitalocean.com/) Optimized Droplets. The information of these droplets is as follows: CPU, Intel^®^ Xeon^®^ Platinum 8168 Processor; Random-access memory, 64 GB; Operating system, Ubuntu 16.04.4×64.

### The effects of population migration and substructure

In this section, we examine how the estimated selection difference 
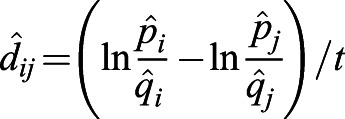
 is affected by population migration and substructure in theory. Here, 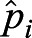 and 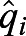 are the observed derived and ancestral allele frequencies in the population *i*, respectively; 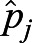 and 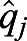 are the observed derived and ancestral allele frequencies in the population *j*, respectively; and *t* is the divergence time from populations *i* and *j* to their most recent common ancestor. We demonstrate that 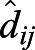 provides a lower bound of selection difference between populations *i* and *j* when migration or substructure exists. We first provide two inequalities that will be used later.

Inequality 1: If *a*>*b*>0, *c*>*d*>0, then *ac*>*bd* and 
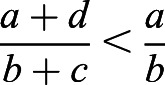
.

Proof: *a*>*b*>0, *c*>*d*>0, then *ac*>*bc*, *bc*>*bd*. Therefore, *ac*>*bd*. Furthermore, *ac*+*ab*>*bd*+*ab*, which is the same as *a*(*b*+*c*)>*b*(*a*+*d*). Therefore, 
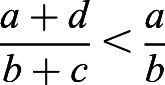
.

Inequality 2: If *a*_1_>0, *a*_2_>0, L, *a*_*n*_>0, 
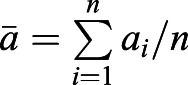
, then


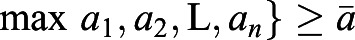
 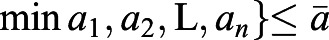.

Proof: Let *a*_max_=max{*a*_1_, *a*_2_, L, *a*_*n*_}, then
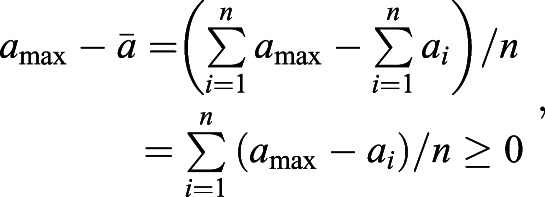
because *a*_max_≥*a*_*i*_.

Let *a*_min_=min{*a*_1_, *a*_2_, …, *a*_*n*_}, then
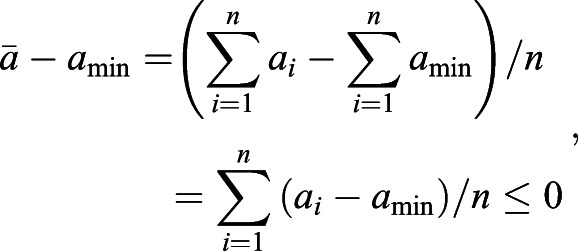
because *a*_min_≤*a*_*i*_.

For the proofs in below, we assume 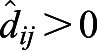 without loss of generality. If 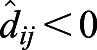, then we can exchange *i* and *j*, and still obtain 
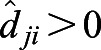
.

### The effect of migration

Suppose there is *α* proportion of individuals in the population *i*, which actually come from the population *j*; also, there is *β* proportion of individuals in the population *j*, which actually come from the population *i*. Here, 
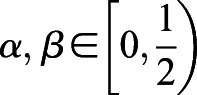
, because we assume migrants should not become the majority of another population. Then we have
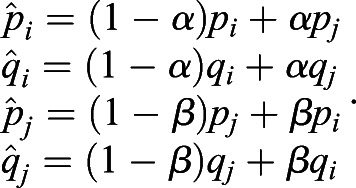
Here, *p*_*i*_ and *q*_*i*_ are the true derived and ancestral allele frequencies in the population *i*, respectively; *p*_*j*_ and *q*_*j*_ are the true derived and ancestral allele frequencies in the population *j*, respectively. Therefore,
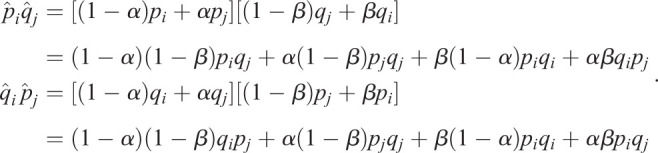
Further, we have
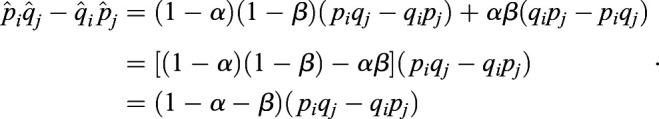


Because 
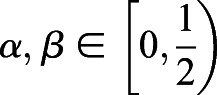
, then 1−*α*−*β*>0; and 
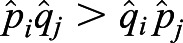
, therefore, *p*_*i*_*q*_*j*_−*q*_*i*_*p*_*j*_>0.

We also have 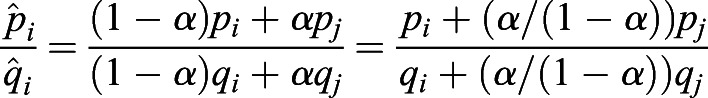. Because *p*_*i*_*q*_*j*_−*q*_*i*_*p*_*j*_>0, we have 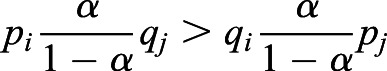. From Inequality 1, we know 
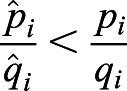
. Similarly, we also have 
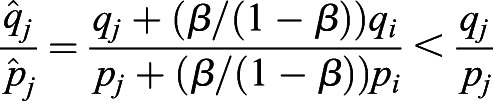
, therefore, 
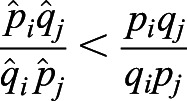
. According to the monotony of the logarithmic function, we have 
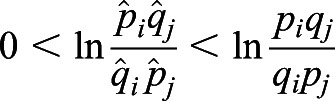
; thus, 
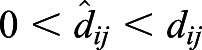
. In other words, if migration exists between populations *i* and *j*, the estimated selection difference is lower than the true value.

### The effect of substructure

Scenario 1: The population *j* has *k* subpopulations.

If the population *j* has *k* subpopulations, then 
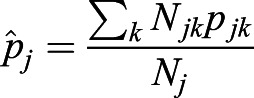
. Here, *p*_*jk*_ is the derived allele frequency in the subpopulation *k* of the population *j*. And *N*_*j*_ is the population size of the population *j*, 
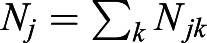
. We denote the minimum of *p*_*jk*_ as min(*p*_*jk*_). Because *q*_*jk*_=1−*p*_*jk*_, then max(*q*_*jk*_)=1−min(*p*_*jk*_). Based on Inequality 2, 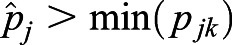, 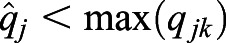. Therefore, 
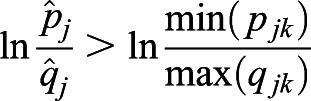
. We have 
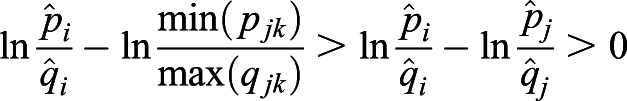
.

Scenario 2: The population *i* has *l* subpopulations.

If the population *i* has *l* subpopulations, then 
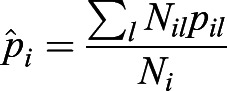
. We denote the maximum of *p*_*il*_ as max(*p*_*il*_). Then 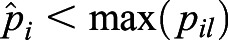, 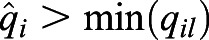. Therefore, 
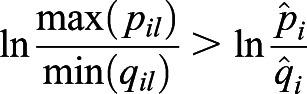
, and we have 
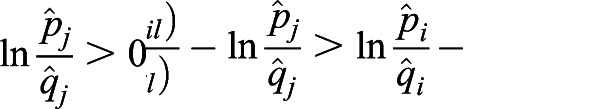
.

Scenario 3: The population *i* has *l* subpopulations, and the population *j* has *k* subpopulations.

Based on scenarios 1 and 2, we have


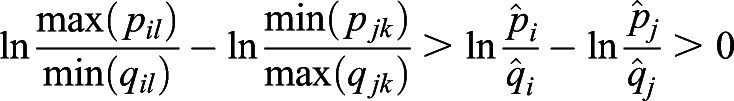
.

In summary, if populations *i* and *j* have subpopulations, and their estimated selection difference is larger than zero, then at least one pair of their subpopulations has selection difference larger than zero. Moreover, the estimated difference is smaller than the largest difference between subpopulations.

## Supplementary Material

Supplementary information
