# Supplementary material for: Dissecting dynamics and differences of selective pressures in the evolution of human pigmentation
Source: Biol Open. 2021 Feb 9;10(2):bio056523. doi: 10.1242/bio.056523 (PMC7888712; doi:10.1242/bio.056523)
Supplement: Supplementary information [file biolopen-10-056523-s1.pdf]

Supplementary Material

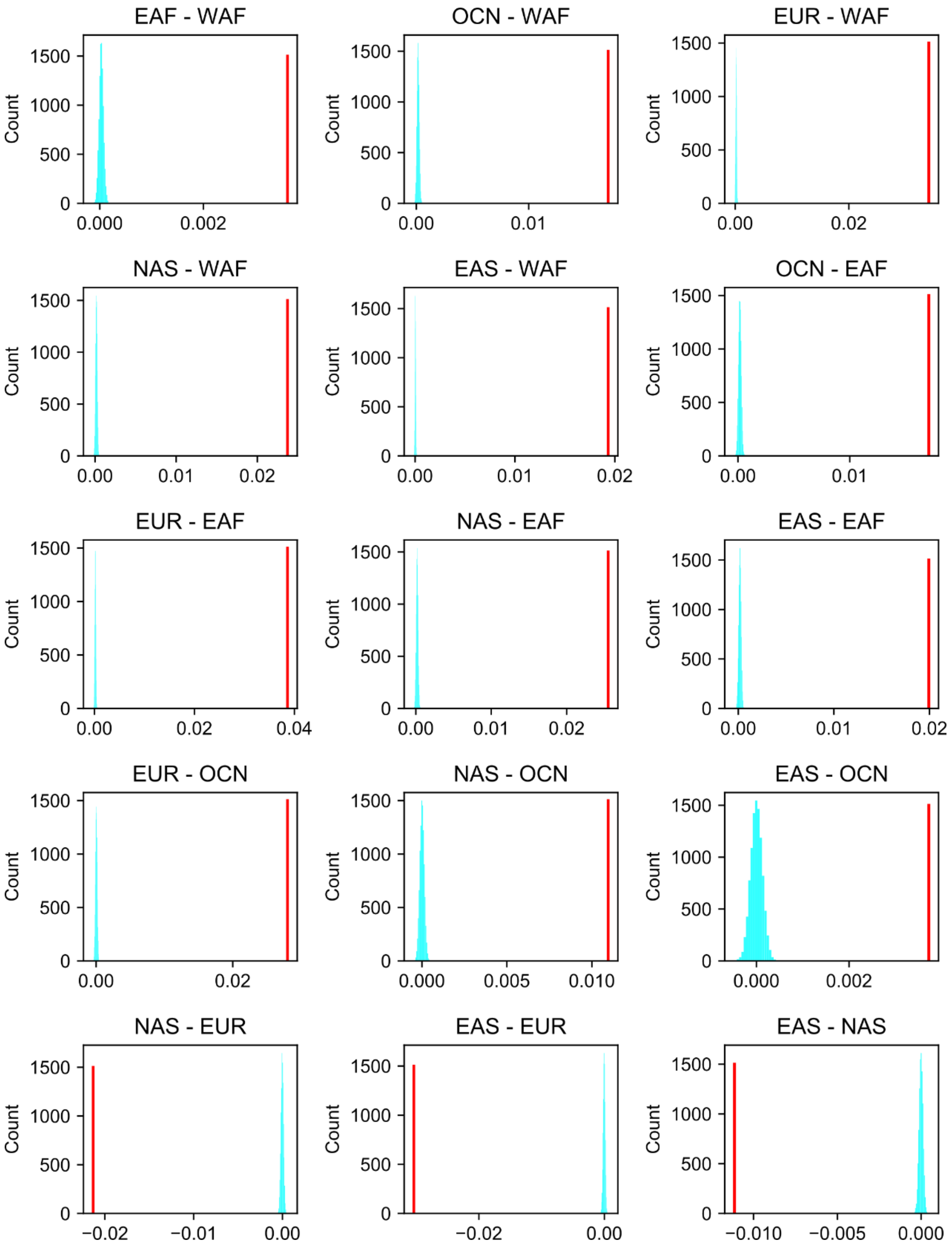

Fig. S1. Significance levels of the selection differences associated with human pigmentation between populations. The blue bars are the empirical distributions of population differences using 10,000 random sets of 30 SNPs in the genomes. The red lines are the selection differences between populations using 30 SNPs associated human pigmentation (Materials and Methods). Population abbreviations: WAF, West Africans; EAF, East Africans; OCN, Oceanians; EUR, Europeans; NAS, North Asians; EAS, East Asians.

Biology Open • Supplementary information

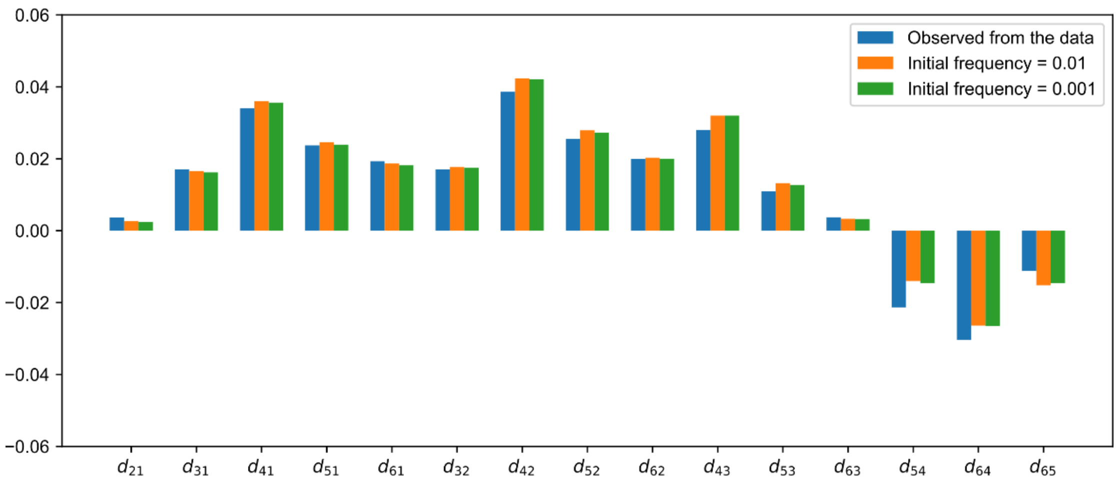

Fig. S2. Comparisons between selection differences from simulation and the data. The selection differences are: d_{21}, differences between East Africans and West Africans; d_{31}, differences between Oceanians and West Africans; d_{41}, differences between Europeans and West Africans; d_{51}, differences between North Asians and West Africans; d_{61}, differences between East Asians and West Africans; d_{32}, differences between Oceanians and East Africans; d_{42}, differences between Europeans and East Africans; d_{52}, differences between North Asians and East Africans; d_{62}, differences between East Asians and East Africans; d_{43}, differences between Europeans and Oceanians; d_{53}, differences between North Asians and Oceanians; d_{63}, differences between East Asians and Oceanians; d_{54}, differences between North Asians and Europeans; d_{64}, differences between East Asians and Europeans; d_{65}, differences between East Asians and North Asians.

Biology Open • Supplementary information

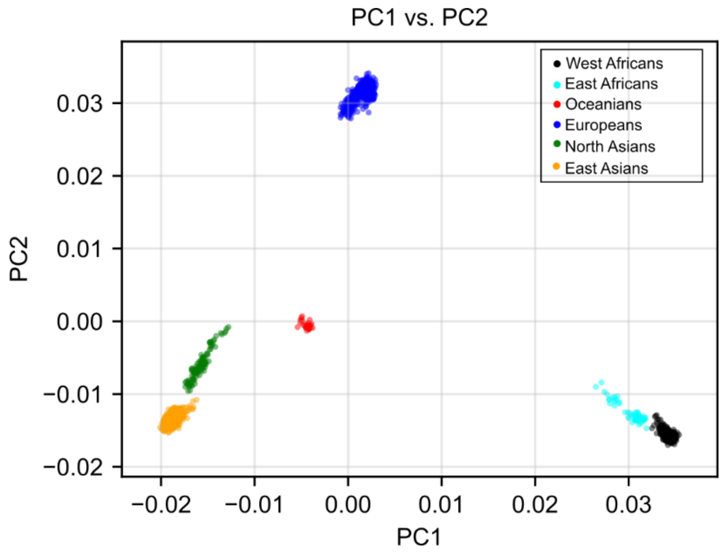

Fig. S3. PCA plot of 2346 samples.

Biology Open • Supplementary information

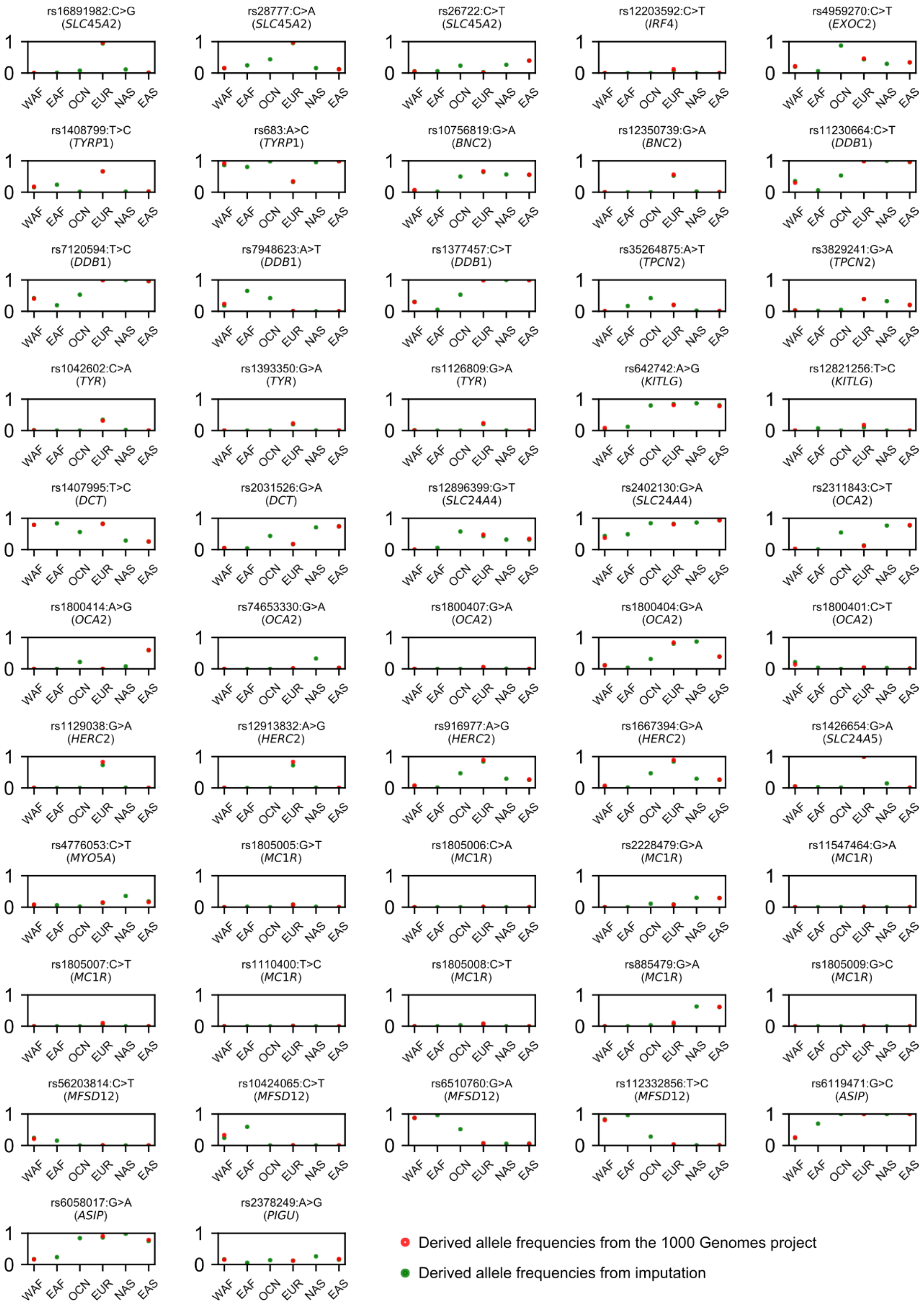

Fig. S4. Derived allele frequencies of candidate SNPs from imputation and the 1000 Genomes project. Population abbreviations: WAF, West Africans; EAF, East Africans; OCN, Oceanians; EUR, Europeans; NAS, North Asians; EAS, East Asians.

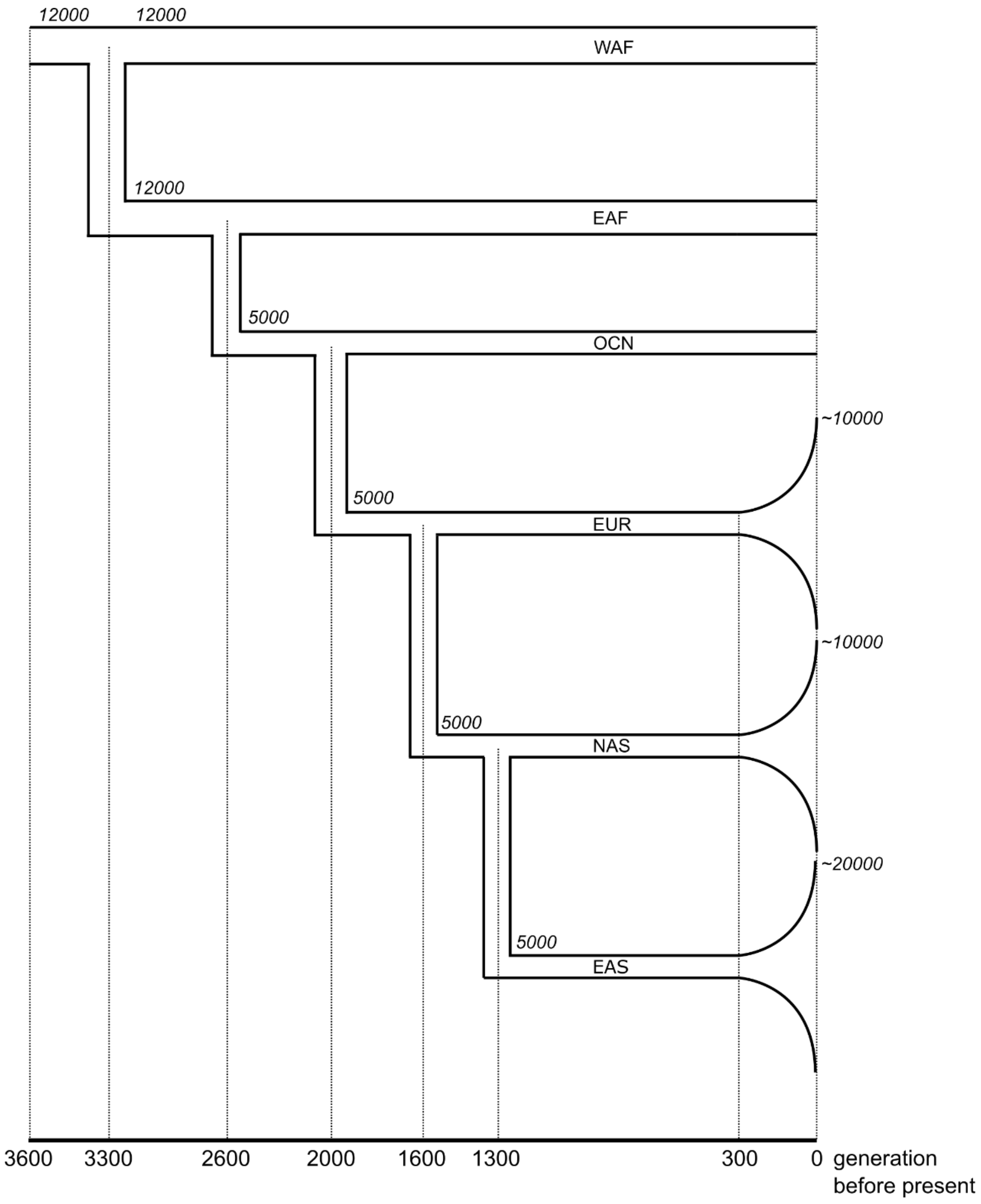

Fig. S5. The demography model for simulation. The italic numbers indicate population sizes at different time periods. Population abbreviations: WAF, West Africans; EAF, East Africans; OCN, Oceanians; EUR, Europeans; NAS, North Asians; EAS, East Asians.

Table S1 Data resources

Dataset	Sample size	Resource	Reference
1KG	2504	http://www.1000genomes.org/	The 1000 Genomes Project Consortium, 2010
HapMap3	1397	http://www.sanger.ac.uk/resources/downloads/human/hapmap3.html	The International HapMap 3 Consortium, 2010
Jew	466	http://evolbio.ut.ee/jew/	Behar, et al., 2010
Afghan	24	http://evolbio.ut.ee/afghan/	Cristofaro, et al., 2013
Sakha	40	http://evolbio.ut.ee/sakha/	Fedorova, et al., 2013
Balkan	70	http://evolbio.ut.ee/balkan/	Kovacevic, et al., 2014
HGDP	1043	ftp://ftp.cephb.fr/hgdp_supp1/	Li, et al., 2008
India	142	http://evolbio.ut.ee/india/	Metspalu, et al., 2011
Ethiopian	235	http://mega.bioanth.cam.ac.uk/data/Ethiopia	Pagani, et al., 2012
Malta	85	http://evolbio.ut.ee/malta/	Raghavan, et al., 2014
Saqqaq	197	http://evolbio.ut.ee/saqqaq/	Rasmussen, et al., 2010
SGVP	268	http://phg.nus.edu.sg/StatGen/public_html/SGVP/download.html	Teo, et al., 2009
NorthernEurasian	369	Request from the authors	Xing, et al., 2013
Caucasus	204	http://evolbio.ut.ee/caucasus/	Yunusbayev, et al., 2012
Turkic	322	http://evolbio.ut.ee/turkic/	Yunusbayev, et al., 2015
Andamanese	10	https://www.ebi.ac.uk/ena/data/view/PRJEB11455	Modal, et al., 2016
EGDP	483	http://evolbio.ut.ee/CGgenomes.html	Pagani, et al., 2016

Biology Open • Supplementary information

Table S2 Population information

Data resource	Population group	Population abbreviation	Sample size
Ethiopian	East Africans	ANU	21
Ethiopian	East Africans	GUM	15
Ethiopian	East Africans	SUD	23
1KG	East Asians	CHN	208
1KG	East Asians	DAI	93
1KG	East Asians	JAP	104
1KG	East Asians	KHV	96
EGDP	East Asians	BUM	1
EGDP	East Asians	DSN	8
EGDP	East Asians	IGO	8
EGDP	East Asians	LBO	4
EGDP	East Asians	LUZ	2
EGDP	East Asians	MUR	8
EGDP	East Asians	VIZ	2
EGDP	East Asians	VTN	10
HapMap3	East Asians	CHN	152
HapMap3	East Asians	JAP	16
HGDP	East Asians	CHN	44
HGDP	East Asians	DAI	10
HGDP	East Asians	JAP	28
HGDP	East Asians	LAH	8
HGDP	East Asians	MIA	10
HGDP	East Asians	NAX	9
HGDP	East Asians	SHE	10
HGDP	East Asians	TUJ	10
HGDP	East Asians	YIZ	10
SGVP	East Asians	CHN	96
1KG	Europeans	CEU	99
1KG	Europeans	FIN	99
1KG	Europeans	GBR	91
Jew	Europeans	BEL	9
Jew	Europeans	HNG	20
Jew	Europeans	LIT	10
Jew	Europeans	RMN	14
Jew	Europeans	RUS	2
EGDP	Europeans	ALB	3
EGDP	Europeans	BEL	4
EGDP	Europeans	COS	4
EGDP	Europeans	CRO	4
EGDP	Europeans	EST	6
EGDP	Europeans	FIN	3
EGDP	Europeans	GER	3
EGDP	Europeans	HNG	2
EGDP	Europeans	ING	3
EGDP	Europeans	KAR	3

Biology Open • Supplementary information

EGDP	Europeans	KOM	2
EGDP	Europeans	LAT	3
EGDP	Europeans	LIT	3
EGDP	Europeans	MOL	2
EGDP	Europeans	MRD	3
EGDP	Europeans	POL	4
EGDP	Europeans	RUS	7
EGDP	Europeans	SWE	2
EGDP	Europeans	UKR	7
EGDP	Europeans	VEP	4
HapMap3	Europeans	CEU	24
Balkan	Europeans	BOS	14
Balkan	Europeans	KSV	9
Balkan	Europeans	MAC	14
Balkan	Europeans	MNT	14
Balkan	Europeans	SER	18
HGDP	Europeans	FRE	28
HGDP	Europeans	ORC	15
HGDP	Europeans	RUS	25
Malta	Europeans	EST	14
Malta	Europeans	RUS	1
NorthernEurasian	Europeans	SLV	25
Turkic	Europeans	GAG	12
Turkic	Europeans	GER	13
Turkic	Europeans	KAR	15
Turkic	Europeans	RUS	33
Turkic	Europeans	VEP	11
EGDP	North Asians	EVK	13
EGDP	North Asians	EVN	8
EGDP	North Asians	NGA	2
EGDP	North Asians	SAK	7
EGDP	North Asians	YAK	1
Sakha	North Asians	DOL	3
Sakha	North Asians	EVN	8
Sakha	North Asians	YAK	1
HGDP	North Asians	YAK	22
Malta	North Asians	DOL	1
Malta	North Asians	EVE	2
Saqqaq	North Asians	DOL	4
Saqqaq	North Asians	EVE	15
Saqqaq	North Asians	NGA	13
Saqqaq	North Asians	YUK	3
Turkic	North Asians	EVE	3
Turkic	North Asians	EVN	3
Turkic	North Asians	NGA	2
Turkic	North Asians	YAK	3
EGDP	Oceanians	KOI	3

Biology Open • Supplementary information

EGDP	Oceanians	KOS	3
HGDP	Oceanians	PAP	16
Andamanese	Oceanians	AND	10
1KG	West Africans	ESN	99
1KG	West Africans	GWD	112
1KG	West Africans	MSL	85
1KG	West Africans	YOR	108
HapMap3	West Africans	YOR	46
HGDP	West Africans	MND	22
HGDP	West Africans	YOR	21

Biology Open • Supplementary information

Table S3 Candidate SNP information

Gene	Chromosome	Position (Hg19)	SNP	Ancestral allele	Derived allele	Pigmentation associated with the derived allele	Reference
SLC45A2	5	33951693	rs16891982	C	G	light	Graf, et al., 2005; Walsh, et al., 2013
SLC45A2	5	33958959	rs28777	C	A	light	Walsh, et al., 2013
SLC45A2	5	33963870	rs26722	C	T	dark	Graf, et al., 2005
IRF4	6	396321	rs12203592	C	T	light	Han, et al., 2008; Praetorius, et al., 2013
EXOC2	6	457748	rs4959270	C	A	light	Walsh, et al., 2013
TYRP1	9	12672097	rs1408799	T	C	light	Sulem, et al., 2008
TYRP1	9	12709305	rs683	A	C	dark	Walsh, et al., 2013
BNC2	9	16858084	rs10756819	G	A	light	Guenther, et al., 2014; Jacobs, et al., 2013
BNC2	9	16885017	rs12350739	G	A	light	Visser, et al., 2012
DDB1	11	61076372	rs11230664	C	T	light	Crawfold, et al., 2017
DDB1	11	61080557	rs7120594	T	C	light	Crawfold, et al., 2017
DDB1	11	61137147	rs7948623	A	T	dark	Crawfold, et al., 2017
DDB1	11	61144652	rs1377457	C	A	light	Crawfold, et al., 2017
TPCN2	11	68846399	rs35264875	A	T	light	Sulem, et al., 2008
TPCN2	11	68855363	rs3829241	G	A	light	Sulem, et al., 2008
TYR	11	88911696	rs1042602	C	A	light	Stokowki, et al., 2007
TYR	11	89011046	rs1393350	G	A	light	Sulem, et al., 2008
TYR	11	89017961	rs1126809	G	A	light	Graf, et al., 2005
KITLG	12	89299746	rs642742	A	G	light	Miller, et al., 2007
KITLG	12	89328335	rs12821256	T	C	light	Sulem, et al., 2004

Biology Open • Supplementary information

DCT	13	95096013	rs1407995	T	C	Unknown	Edwards, et al., 2010; Ainger, et al., 2017
DCT	13	95100841	rs2031526	G	A	Unknown	Edwards, et al., 2010; Ainger, et al., 2017
SLC24A4	14	92773663	rs12896399	G	T	light	Sulem, et al., 2007
SLC24A4	14	92801203	rs2402130	G	A	light	Walsh, et al., 2013
OCA2	15	28183354	rs2311843	C	T	Unknown	Anno, et al., 2008
OCA2	15	28197037	rs1800414	A	G	light	Edwards, et al., 2010; Yang, et al., 2016; Murray, et al. 2015
OCA2	15	28228553	rs74653330	G	A	light	Murray, et al., 2015
OCA2	15	28230318	rs1800407	G	A	light	Branicki, et al., 2009; Branicki, et al., 2011
OCA2	15	28235773	rs1800404	G	A	light	Crawfold, et al., 2017
OCA2	15	28260053	rs1800401	C	T	dark	Rebbeck, et al., 2002
HERC2	15	28356859	rs1129038	G	A	light	Donnelly, et al., 2012; Eiberg, et al., 2008
HERC2	15	28365618	rs12913832	A	G	light	Sulem, et al., 2008; Sturm, et al., 2008
HERC2	15	28513364	rs916977	A	G	light	Kayser, et al., 2008; Donnelly, et al., 2012
HERC2	15	28530182	rs1667394	G	A	light	Sulem, et al., 2007; Donnelly, et al., 2012
SLC24A5	15	48426484	rs1426654	G	A	light	Lamason, et al., 2005
MYO5A	15	52816195	rs4776053	C	T	Unknown	Anno, et al., 2008
MC1R	16	89985844	rs1805005	G	T	light	Walsh, et al., 2013
MC1R	16	89985918	rs1805006	C	A	light	Walsh, et al., 2013
MC1R	16	89985940	rs2228479	G	A	light	Walsh, et al., 2013
MC1R	16	89986091	rs11547464	G	A	light	Walsh, et al., 2013
MC1R	16	89986117	rs1805007	C	T	light	Walsh, et al., 2013
MC1R	16	89986130	rs1110400	T	C	light	Walsh, et al., 2013
MC1R	16	89986144	rs1805008	C	T	light	Walsh, et al., 2013
MC1R	16	89986154	rs885479	G	A	light	Walsh, et al., 2013
MC1R	16	89986546	rs1805009	G	C	light	Walsh, et al., 2013

Biology Open • Supplementary information

MFSD12	19	3544892	rs56203814	C	T	dark	Crawfold, et al., 2017
MFSD12	19	3545022	rs10424065	C	T	dark	Crawfold, et al., 2017
MFSD12	19	3565253	rs6510760	G	A	dark	Crawfold, et al., 2017
MFSD12	19	3565599	rs112332856	T	C	dark	Crawfold, et al., 2017
ASIP	20	32785212	rs6119471	G	C	light	Hart, et al., 2013
ASIP	20	32856998	rs6058017	G	A	light	Bonilla, et al., 2005
PIGU	20	33218090	rs2378249	A	G	light	Branicki, et al., 2011

Table S4 Selection differences on the 42 selected SNPs

Gene	SNP ID	Ancestral allele	Derived allele	Population1	Population2	Selection difference (Population1 - Population2)	Std	*p*-value
SLC45A2	rs16891982	C	G	Europeans	NorthAsians	0.003181	7.47E-04	**2.10E-05**
SLC45A2	rs28777	C	A	Europeans	NorthAsians	0.003107	7.46E-04	**3.10E-05**
SLC45A2	rs26722	C	T	Europeans	NorthAsians	-0.001767	7.47E-04	**0.017939**
EXOC2	rs4959270	C	A	Europeans	NorthAsians	4.12E-04	7.38E-04	0.576675
TYRP1	rs1408799	T	C	Europeans	NorthAsians	0.00287	7.90E-04	**2.82E-04**
TYRP1	rs683	A	C	Europeans	NorthAsians	-0.002354	7.60E-04	**0.001943**
BNC2	rs10756819	G	A	Europeans	NorthAsians	2.33E-04	7.37E-04	0.752243
BNC2	rs12350739	G	A	Europeans	NorthAsians	0.002475	7.85E-04	**0.00161**
DDB1	rs11230664	C	T	Europeans	NorthAsians	-8.56E-04	0.001162	0.461178
DDB1	rs7120594	T	C	Europeans	NorthAsians	-5.17E-04	0.001172	0.658962
DDB1	rs7948623	A	T	Europeans	NorthAsians	4.21E-04	0.001176	0.720668
DDB1	rs1377457	C	A	Europeans	NorthAsians	-7.42E-04	0.001165	0.524412
TPCN2	rs35264875	A	T	Europeans	NorthAsians	0.00152	7.85E-04	0.052919
TPCN2	rs3829241	G	A	Europeans	NorthAsians	1.79E-04	7.38E-04	0.808351
TYR	rs1042602	C	A	Europeans	NorthAsians	0.002002	7.90E-04	**0.011305**
TYR	rs1393350	G	A	Europeans	NorthAsians	0.0023	8.94E-04	**0.010076**
TYR	rs1126809	G	A	Europeans	NorthAsians	0.002329	8.94E-04	**0.009156**
KITLG	rs642742	A	G	Europeans	NorthAsians	-1.94E-04	7.43E-04	0.794378
KITLG	rs12821256	T	C	Europeans	NorthAsians	0.002653	0.001149	**0.020951**
DCT	rs1407995	T	C	Europeans	NorthAsians	0.001528	7.38E-04	**0.038476**
DCT	rs2031526	G	A	Europeans	NorthAsians	-0.001525	7.38E-04	**0.038867**
SLC24A4	rs12896399	G	T	Europeans	NorthAsians	3.51E-04	7.38E-04	0.634377
SLC24A4	rs2402130	G	A	Europeans	NorthAsians	-2.25E-04	7.43E-04	0.761965
OCA2	rs2311843	C	T	Europeans	NorthAsians	-0.001944	7.40E-04	**0.008596**
OCA2	rs1800414	A	G	Europeans	NorthAsians	-0.002739	9.05E-04	**0.002475**
OCA2	rs1800404	C	T	Europeans	NorthAsians	-2.52E-04	7.43E-04	0.73489

OCA2	rs1800401	C	A	Europeans	NorthAsians	2.58E-04	7.81E-04	0.741106
HERC2	rs1129038	G	A	Europeans	NorthAsians	0.003579	8.33E-04	**1.80E-05**
HERC2	rs12913832	A	G	Europeans	NorthAsians	0.003566	8.33E-04	**1.90E-05**
HERC2	rs916977	A	G	Europeans	NorthAsians	0.001695	7.39E-04	**0.02177**
HERC2	rs1667394	G	A	Europeans	NorthAsians	0.001684	7.39E-04	**0.022651**
SLC24A5	rs1426654	G	A	Europeans	NorthAsians	0.004135	7.65E-04	**0**
MYO5A	rs4776053	C	T	Europeans	NorthAsians	-7.63E-04	7.38E-04	0.301197
MC1R	rs2228479	G	A	Europeans	NorthAsians	-9.75E-04	7.40E-04	0.187511
MC1R	rs885479	G	A	Europeans	NorthAsians	-0.001829	7.39E-04	**0.013324**
MFSD12	rs56203814	C	T	Europeans	NorthAsians	2.40E-04	0.001185	0.839641
MFSD12	rs10424065	C	T	Europeans	NorthAsians	2.40E-04	0.001185	0.839641
MFSD12	rs6510760	G	A	Europeans	NorthAsians	1.39E-04	7.58E-04	0.85424
MFSD12	rs112332856	T	C	Europeans	NorthAsians	9.48E-04	8.98E-04	0.291189
ASIP	rs6119471	G	C	Europeans	NorthAsians	0.001135	0.001449	0.433361
ASIP	rs6058017	G	A	Europeans	NorthAsians	-0.001192	7.91E-04	0.132079
PIGU	rs2378249	A	G	Europeans	NorthAsians	-5.74E-04	7.39E-04	0.437671
SLC45A2	rs16891982	C	G	Europeans	Oceanians	0.002754	6.98E-04	**8.00E-05**
SLC45A2	rs28777	C	A	Europeans	Oceanians	0.001774	6.71E-04	**0.008209**
SLC45A2	rs26722	C	T	Europeans	Oceanians	-0.001337	6.78E-04	**0.048735**
EXOC2	rs4959270	C	A	Europeans	Oceanians	-0.001082	6.83E-04	0.113007
TYRP1	rs1408799	T	C	Europeans	Oceanians	0.002211	7.75E-04	**0.00434**
TYRP1	rs683	A	C	Europeans	Oceanians	-0.002211	7.75E-04	**0.00434**
BNC2	rs10756819	G	A	Europeans	Oceanians	3.18E-04	6.68E-04	0.633414
BNC2	rs12350739	G	A	Europeans	Oceanians	0.002508	9.67E-04	**0.009475**
DDB1	rs11230664	C	T	Europeans	Oceanians	0.002313	6.83E-04	**7.03E-04**
DDB1	rs7120594	T	C	Europeans	Oceanians	0.002585	6.94E-04	**1.94E-04**
DDB1	rs7948623	A	T	Europeans	Oceanians	-0.002567	6.98E-04	**2.36E-04**
DDB1	rs1377457	C	A	Europeans	Oceanians	0.002405	6.86E-04	**4.53E-04**
TPCN2	rs35264875	A	T	Europeans	Oceanians	-5.25E-04	6.68E-04	0.431752

Biology Open • Supplementary information

TPCN2	rs3829241	G	A	Europeans	Oceanians	0.001206	7.11E-04	0.090003
TYR	rs1042602	C	A	Europeans	Oceanians	0.002074	9.67E-04	**0.031884**
TYR	rs1393350	G	A	Europeans	Oceanians	0.001756	9.67E-04	0.069267
TYR	rs1126809	G	A	Europeans	Oceanians	0.00178	9.67E-04	0.065616
KITLG	rs642742	A	G	Europeans	Oceanians	1.05E-04	6.74E-04	0.876062
KITLG	rs12821256	T	C	Europeans	Oceanians	0.001487	9.67E-04	0.124028
DCT	rs1407995	T	C	Europeans	Oceanians	6.48E-04	6.68E-04	0.332199
DCT	rs2031526	G	A	Europeans	Oceanians	-6.46E-04	6.68E-04	0.334037
SLC24A4	rs12896399	G	T	Europeans	Oceanians	-2.53E-04	6.68E-04	0.704428
SLC24A4	rs2402130	G	A	Europeans	Oceanians	-9.90E-05	6.78E-04	0.884527
OCA2	rs2311843	C	T	Europeans	Oceanians	-0.001052	6.68E-04	0.115666
OCA2	rs1800414	A	G	Europeans	Oceanians	-0.002783	7.87E-04	**4.05E-04**
OCA2	rs1800404	C	T	Europeans	Oceanians	0.001137	6.70E-04	0.08982
OCA2	rs1800401	C	T	Europeans	Oceanians	8.27E-04	9.69E-04	0.393278
HERC2	rs1129038	G	A	Europeans	Oceanians	0.003037	9.67E-04	**0.001678**
HERC2	rs12913832	A	G	Europeans	Oceanians	0.003027	9.67E-04	**0.001739**
HERC2	rs916977	A	G	Europeans	Oceanians	9.80E-04	6.68E-04	0.142565
HERC2	rs1667394	G	A	Europeans	Oceanians	9.71E-04	6.68E-04	0.14624
SLC24A5	rs1426654	G	A	Europeans	Oceanians	0.004289	7.89E-04	**0**
MYO5A	rs4776053	C	T	Europeans	Oceanians	9.60E-04	7.76E-04	0.215663
MC1R	rs2228479	G	A	Europeans	Oceanians	-1.59E-04	6.87E-04	0.816976
MC1R	rs885479	G	A	Europeans	Oceanians	4.12E-04	7.32E-04	0.57365
MFSD12	rs56203814	C	T	Europeans	Oceanians	-4.43E-04	9.95E-04	0.655763
MFSD12	rs10424065	C	T	Europeans	Oceanians	-4.43E-04	9.95E-04	0.655763
MFSD12	rs6510760	G	A	Europeans	Oceanians	-0.001365	6.69E-04	**0.041418**
MFSD12	rs112332856	T	C	Europeans	Oceanians	-0.001282	6.75E-04	0.057377
ASIP	rs6119471	G	C	Europeans	Oceanians	0.001543	0.001197	0.197389
ASIP	rs6058017	G	A	Europeans	Oceanians	1.57E-04	6.79E-04	0.816854
PIGU	rs2378249	A	G	Europeans	Oceanians	-6.90E-05	6.81E-04	0.9196

Biology Open • Supplementary information

SLC45A2	rs16891982	C	G	Europeans	EastAsians	0.004804	7.53E-04	**0**
SLC45A2	rs28777	C	A	Europeans	EastAsians	0.003301	7.40E-04	**8.00E-06**
SLC45A2	rs26722	C	T	Europeans	EastAsians	-0.002147	7.43E-04	**0.00388**
EXOC2	rs4959270	C	A	Europeans	EastAsians	2.80E-04	7.35E-04	0.70332
TYRP1	rs1408799	T	C	Europeans	EastAsians	0.002875	7.42E-04	**1.06E-04**
TYRP1	rs683	A	C	Europeans	EastAsians	-0.003298	7.48E-04	**1.10E-05**
BNC2	rs10756819	G	A	Europeans	EastAsians	2.60E-04	7.35E-04	0.723261
BNC2	rs12350739	G	A	Europeans	EastAsians	0.003316	7.58E-04	**1.20E-05**
DDB1	rs11230664	C	T	Europeans	EastAsians	8.84E-04	7.60E-04	0.244405
DDB1	rs7120594	T	C	Europeans	EastAsians	0.001224	7.75E-04	0.114356
DDB1	rs7948623	A	T	Europeans	EastAsians	5.27E-04	8.46E-04	0.533572
DDB1	rs1377457	C	A	Europeans	EastAsians	-2.40E-05	7.80E-04	0.97525
TPCN2	rs35264875	A	T	Europeans	EastAsians	0.002125	7.51E-04	**0.004686**
TPCN2	rs3829241	G	A	Europeans	EastAsians	5.75E-04	7.35E-04	0.43454
TYR	rs1042602	C	A	Europeans	EastAsians	0.003703	8.34E-04	**9.00E-06**
TYR	rs1393350	G	A	Europeans	EastAsians	0.003095	8.07E-04	**1.26E-04**
TYR	rs1126809	G	A	Europeans	EastAsians	0.003655	8.95E-04	**4.40E-05**
KITLG	rs642742	A	G	Europeans	EastAsians	1.58E-04	7.36E-04	0.829732
KITLG	rs12821256	T	C	Europeans	EastAsians	0.003289	8.95E-04	**2.38E-04**
DCT	rs1407995	T	C	Europeans	EastAsians	0.001625	7.36E-04	**0.027173**
DCT	rs2031526	G	A	Europeans	EastAsians	-0.001627	7.36E-04	**0.026976**
SLC24A4	rs12896399	G	T	Europeans	EastAsians	3.12E-04	7.35E-04	0.67082
SLC24A4	rs2402130	G	A	Europeans	EastAsians	-7.69E-04	7.37E-04	0.296629
OCA2	rs2311843	C	T	Europeans	EastAsians	-0.00198	7.36E-04	**0.007154**
OCA2	rs1800414	A	G	Europeans	EastAsians	-0.004515	8.94E-04	**0**
OCA2	rs1800404	C	T	Europeans	EastAsians	0.001211	7.35E-04	0.099601
OCA2	rs1800401	C	T	Europeans	EastAsians	0.001064	7.57E-04	0.160263
HERC2	rs1129038	G	A	Europeans	EastAsians	0.004539	7.92E-04	**0**
HERC2	rs12913832	A	G	Europeans	EastAsians	0.004684	8.07E-04	**0**

Biology Open • Supplementary information

HERC2	rs916977	A	G	Europeans	EastAsians	0.001788	7.36E-04	**0.015136**
HERC2	rs1667394	G	A	Europeans	EastAsians	0.001778	7.36E-04	**0.015681**
SLC24A5	rs1426654	G	A	Europeans	EastAsians	0.005774	7.69E-04	**0**
MYO5A	rs4776053	C	T	Europeans	EastAsians	-1.45E-04	7.36E-04	0.844254
MC1R	rs2228479	G	A	Europeans	EastAsians	-9.51E-04	7.37E-04	0.196783
MC1R	rs885479	G	A	Europeans	EastAsians	-0.001785	7.37E-04	**0.015392**
MFSD12	rs56203814	C	T	Europeans	EastAsians	0.001563	0.001186	0.18755
MFSD12	rs10424065	C	T	Europeans	EastAsians	8.76E-04	9.41E-04	0.351953
MFSD12	rs6510760	G	A	Europeans	EastAsians	1.37E-04	7.40E-04	0.85293
MFSD12	rs112332856	T	C	Europeans	EastAsians	0.001151	7.69E-04	0.134437
ASIP	rs6119471	G	C	Europeans	EastAsians	-1.88E-04	0.00145	0.896809
ASIP	rs6058017	G	A	Europeans	EastAsians	4.96E-04	7.36E-04	0.500451
PIGU	rs2378249	A	G	Europeans	EastAsians	-2.14E-04	7.36E-04	0.770841
SLC45A2	rs16891982	C	G	Europeans	EastAfricans	0.002845	6.31E-04	**6.00E-06**
SLC45A2	rs28777	C	A	Europeans	EastAfricans	0.001699	5.53E-04	**0.002098**
SLC45A2	rs26722	C	T	Europeans	EastAfricans	-4.21E-04	5.68E-04	0.459218
EXOC2	rs4959270	C	A	Europeans	EastAfricans	9.79E-04	5.64E-04	0.08264
TYRP1	rs1408799	T	C	Europeans	EastAfricans	7.12E-04	5.50E-04	0.195475
TYRP1	rs683	A	C	Europeans	EastAfricans	-8.09E-04	5.51E-04	0.142307
BNC2	rs10756819	G	A	Europeans	EastAfricans	0.001721	5.97E-04	**0.003941**
BNC2	rs12350739	G	A	Europeans	EastAfricans	0.002164	7.70E-04	**0.004947**
DDB1	rs11230664	C	T	Europeans	EastAfricans	0.002891	5.75E-04	**0**
DDB1	rs7120594	T	C	Europeans	EastAfricans	0.002582	5.70E-04	**6.00E-06**
DDB1	rs7948623	A	T	Europeans	EastAfricans	-0.002338	5.71E-04	**4.20E-05**
DDB1	rs1377457	C	A	Europeans	EastAfricans	0.003024	5.80E-04	**0**
TPCN2	rs35264875	A	T	Europeans	EastAfricans	8.60E-05	5.52E-04	0.876402
TPCN2	rs3829241	G	A	Europeans	EastAfricans	0.001304	5.97E-04	**0.028911**
TYR	rs1042602	C	A	Europeans	EastAfricans	0.001831	7.70E-04	**0.017436**
TYR	rs1393350	G	A	Europeans	EastAfricans	0.001586	7.70E-04	**0.039444**

Biology Open • Supplementary information

TYR	rs1126809	G	A	Europeans	EastAfricans	0.001604	7.70E-04	**0.037246**
KITLG	rs642742	A	G	Europeans	EastAfricans	0.001378	5.55E-04	**0.013057**
KITLG	rs12821256	T	C	Europeans	EastAfricans	2.86E-04	5.62E-04	0.611079
DCT	rs1407995	T	C	Europeans	EastAfricans	-4.00E-05	5.53E-04	0.942694
DCT	rs2031526	G	A	Europeans	EastAfricans	6.06E-04	5.72E-04	0.289335
SLC24A4	rs12896399	G	T	Europeans	EastAfricans	9.89E-04	5.64E-04	0.079557
SLC24A4	rs2402130	G	A	Europeans	EastAfricans	5.86E-04	5.49E-04	0.285706
OCA2	rs2311843	C	T	Europeans	EastAfricans	9.39E-04	6.29E-04	0.135776
OCA2	rs1800414	A	G	Europeans	EastAfricans	-5.29E-04	8.31E-04	0.524534
OCA2	rs1800404	C	T	Europeans	EastAfricans	0.001814	5.75E-04	**0.001596**
OCA2	rs1800401	C	T	Europeans	EastAfricans	1.30E-05	5.77E-04	0.982
HERC2	rs1129038	G	A	Europeans	EastAfricans	0.002572	7.70E-04	**8.41E-04**
HERC2	rs12913832	A	G	Europeans	EastAfricans	0.002564	7.70E-04	**8.72E-04**
HERC2	rs916977	A	G	Europeans	EastAfricans	0.002182	5.97E-04	**2.60E-04**
HERC2	rs1667394	G	A	Europeans	EastAfricans	0.002175	5.97E-04	**2.72E-04**
SLC24A5	rs1426654	G	A	Europeans	EastAfricans	0.003205	5.94E-04	**0**
MYO5A	rs4776053	C	T	Europeans	EastAfricans	3.64E-04	5.65E-04	0.518997
MC1R	rs2228479	G	A	Europeans	EastAfricans	0.001173	7.71E-04	0.128136
MC1R	rs885479	G	A	Europeans	EastAfricans	0.001183	7.71E-04	0.124649
MFSD12	rs56203814	C	T	Europeans	EastAfricans	-0.001548	5.81E-04	**0.007776**
MFSD12	rs10424065	C	T	Europeans	EastAfricans	-0.002352	5.78E-04	**4.60E-05**
MFSD12	rs6510760	G	A	Europeans	EastAfricans	-0.002269	5.76E-04	**8.10E-05**
MFSD12	rs112332856	T	C	Europeans	EastAfricans	-0.00259	5.77E-04	**7.00E-06**
ASIP	rs6119471	G	C	Europeans	EastAfricans	0.002737	7.73E-04	**3.98E-04**
ASIP	rs6058017	G	A	Europeans	EastAfricans	0.001219	5.51E-04	**0.026928**
PIGU	rs2378249	A	G	Europeans	EastAfricans	3.14E-04	5.65E-04	0.578311
SLC45A2	rs16891982	C	G	Europeans	WestAfricans	0.00263	4.79E-04	**0**
SLC45A2	rs28777	C	A	Europeans	WestAfricans	0.001508	4.52E-04	**8.40E-04**
SLC45A2	rs26722	C	T	Europeans	WestAfricans	-2.48E-04	4.55E-04	0.585986

Biology Open • Supplementary information

EXOC2	rs4959270	C	A	Europeans	WestAfricans	3.28E-04	4.50E-04	0.465309
TYRP1	rs1408799	T	C	Europeans	WestAfricans	6.83E-04	4.50E-04	0.128978
TYRP1	rs683	A	C	Europeans	WestAfricans	-8.97E-04	4.50E-04	**0.046465**
BNC2	rs10756819	G	A	Europeans	WestAfricans	9.96E-04	4.51E-04	**0.027057**
BNC2	rs12350739	G	A	Europeans	WestAfricans	0.00186	4.88E-04	**1.39E-04**
DDB1	rs11230664	C	T	Europeans	WestAfricans	0.001682	4.58E-04	**2.38E-04**
DDB1	rs7120594	T	C	Europeans	WestAfricans	0.001719	4.64E-04	**2.09E-04**
DDB1	rs7948623	A	T	Europeans	WestAfricans	-0.001286	4.66E-04	**0.005809**
DDB1	rs1377457	C	A	Europeans	WestAfricans	0.001757	4.59E-04	**1.31E-04**
TPCN2	rs35264875	A	T	Europeans	WestAfricans	0.001083	4.64E-04	**0.019467**
TPCN2	rs3829241	G	A	Europeans	WestAfricans	9.58E-04	4.53E-04	0.034415
TYR	rs1042602	C	A	Europeans	WestAfricans	0.001495	4.77E-04	**0.001739**
TYR	rs1393350	G	A	Europeans	WestAfricans	0.001893	6.21E-04	**0.002296**
TYR	rs1126809	G	A	Europeans	WestAfricans	0.001317	4.78E-04	**0.005828**
KITLG	rs642742	A	G	Europeans	WestAfricans	0.001239	4.51E-04	**0.005976**
KITLG	rs12821256	T	C	Europeans	WestAfricans	0.00173	6.21E-04	**0.005338**
DCT	rs1407995	T	C	Europeans	WestAfricans	6.70E-05	4.50E-04	0.881067
DCT	rs2031526	G	A	Europeans	WestAfricans	3.96E-04	4.51E-04	0.380466
SLC24A4	rs12896399	G	T	Europeans	WestAfricans	0.001753	4.88E-04	**3.32E-04**
SLC24A4	rs2402130	G	A	Europeans	WestAfricans	5.91E-04	4.50E-04	0.188372
OCA2	rs2311843	C	T	Europeans	WestAfricans	5.25E-04	4.54E-04	0.246673
OCA2	rs1800414	A	G	Europeans	WestAfricans	2.26E-04	6.68E-04	0.734638
OCA2	rs1800404	C	T	Europeans	WestAfricans	0.001079	4.50E-04	**0.016588**
OCA2	rs1800401	C	T	Europeans	WestAfricans	-4.49E-04	4.51E-04	0.320382
HERC2	rs1129038	G	A	Europeans	WestAfricans	0.00197	4.69E-04	**2.70E-05**
HERC2	rs12913832	A	G	Europeans	WestAfricans	0.001964	4.69E-04	**2.80E-05**
HERC2	rs916977	A	G	Europeans	WestAfricans	0.001317	4.51E-04	**0.003482**
HERC2	rs1667394	G	A	Europeans	WestAfricans	0.001354	4.51E-04	**0.002681**
SLC24A5	rs1426654	G	A	Europeans	WestAfricans	0.002367	4.60E-04	**0**

Biology Open • Supplementary information

MYO5A	rs4776053	C	T	Europeans	WestAfricans	1.97E-04	4.51E-04	0.661346
MC1R	rs2228479	G	A	Europeans	WestAfricans	0.001567	6.21E-04	**0.011649**
MC1R	rs885479	G	A	Europeans	WestAfricans	0.001576	6.21E-04	**0.011201**
MFSD12	rs56203814	C	T	Europeans	WestAfricans	-0.001347	4.72E-04	**0.004295**
MFSD12	rs10424065	C	T	Europeans	WestAfricans	-0.001503	4.71E-04	**0.001434**
MFSD12	rs6510760	G	A	Europeans	WestAfricans	-0.001407	4.51E-04	**0.001801**
MFSD12	rs112332856	T	C	Europeans	WestAfricans	-0.001501	4.52E-04	**8.93E-04**
ASIP	rs6119471	G	C	Europeans	WestAfricans	0.002735	6.21E-04	**1.10E-05**
ASIP	rs6058017	G	A	Europeans	WestAfricans	0.001095	4.50E-04	**0.015015**
PIGU	rs2378249	A	G	Europeans	WestAfricans	-8.40E-05	4.50E-04	0.852618
SLC45A2	rs16891982	C	G	NorthAsians	Oceanians	2.09E-04	6.90E-04	0.761863
SLC45A2	rs28777	C	A	NorthAsians	Oceanians	-7.11E-04	6.59E-04	0.280548
SLC45A2	rs26722	C	T	NorthAsians	Oceanians	7.70E-05	6.62E-04	0.907271
EXOC2	rs4959270	C	A	NorthAsians	Oceanians	-0.001411	6.72E-04	**0.03568**
TYRP1	rs1408799	T	C	NorthAsians	Oceanians	-8.50E-05	7.99E-04	0.915284
TYRP1	rs683	A	C	NorthAsians	Oceanians	-3.28E-04	7.79E-04	0.67392
BNC2	rs10756819	G	A	NorthAsians	Oceanians	1.32E-04	6.56E-04	0.840146
BNC2	rs12350739	G	A	NorthAsians	Oceanians	5.27E-04	9.83E-04	0.591614
DDB1	rs11230664	C	T	NorthAsians	Oceanians	0.002999	9.63E-04	**0.001845**
DDB1	rs7120594	T	C	NorthAsians	Oceanians	0.002999	9.63E-04	**0.001845**
DDB1	rs7948623	A	T	NorthAsians	Oceanians	-0.002904	9.63E-04	**0.00257**
DDB1	rs1377457	C	A	NorthAsians	Oceanians	0.002999	9.63E-04	**0.001845**
TPCN2	rs35264875	A	T	NorthAsians	Oceanians	-0.001741	6.91E-04	**0.011742**
TPCN2	rs3829241	G	A	NorthAsians	Oceanians	0.001063	7.01E-04	0.12938
TYR	rs1042602	C	A	NorthAsians	Oceanians	4.72E-04	9.85E-04	0.631774
TYR	rs1393350	G	A	NorthAsians	Oceanians	-8.40E-05	0.00104	0.935849
TYR	rs1126809	G	A	NorthAsians	Oceanians	-8.40E-05	0.00104	0.935849
KITLG	rs642742	A	G	NorthAsians	Oceanians	2.60E-04	6.66E-04	0.696333
KITLG	rs12821256	T	C	NorthAsians	Oceanians	-6.35E-04	0.00119	0.593386

DCT	rs1407995	T	C	NorthAsians	Oceanians	-5.75E-04	6.57E-04	0.381764
DCT	rs2031526	G	A	NorthAsians	Oceanians	5.75E-04	6.57E-04	0.381764
SLC24A4	rs12896399	G	T	NorthAsians	Oceanians	-5.34E-04	6.57E-04	0.416225
SLC24A4	rs2402130	G	A	NorthAsians	Oceanians	8.10E-05	6.70E-04	0.903342
OCA2	rs2311843	C	T	NorthAsians	Oceanians	5.03E-04	6.58E-04	0.444072
OCA2	rs1800414	A	G	NorthAsians	Oceanians	-5.92E-04	6.70E-04	0.376725
OCA2	rs1800404	C	T	NorthAsians	Oceanians	0.001338	6.62E-04	**0.04329**
OCA2	rs1800401	C	T	NorthAsians	Oceanians	6.21E-04	9.78E-04	0.525882
HERC2	rs1129038	G	A	NorthAsians	Oceanians	1.74E-04	0.001008	0.862992
HERC2	rs12913832	A	G	NorthAsians	Oceanians	1.74E-04	0.001008	0.862992
HERC2	rs916977	A	G	NorthAsians	Oceanians	-3.76E-04	6.57E-04	0.567236
HERC2	rs1667394	G	A	NorthAsians	Oceanians	-3.76E-04	6.57E-04	0.567236
SLC24A5	rs1426654	G	A	NorthAsians	Oceanians	9.81E-04	7.68E-04	0.201703
MYO5A	rs4776053	C	T	NorthAsians	Oceanians	0.001571	7.65E-04	**0.04014**
MC1R	rs2228479	G	A	NorthAsians	Oceanians	6.21E-04	6.75E-04	0.357846
MC1R	rs885479	G	A	NorthAsians	Oceanians	0.001875	7.21E-04	**0.009264**
MFSD12	rs56203814	C	T	NorthAsians	Oceanians	-6.35E-04	0.00119	0.593386
MFSD12	rs10424065	C	T	NorthAsians	Oceanians	-6.35E-04	0.00119	0.593386
MFSD12	rs6510760	G	A	NorthAsians	Oceanians	-0.001476	6.69E-04	**0.027402**
MFSD12	rs112332856	T	C	NorthAsians	Oceanians	-0.002041	7.73E-04	**0.008293**
ASIP	rs6119471	G	C	NorthAsians	Oceanians	6.35E-04	0.00119	0.593386
ASIP	rs6058017	G	A	NorthAsians	Oceanians	0.001111	7.05E-04	0.115076
PIGU	rs2378249	A	G	NorthAsians	Oceanians	3.90E-04	6.70E-04	0.560041
SLC45A2	rs16891982	C	G	NorthAsians	EastAsians	0.001997	5.71E-04	**4.71E-04**
SLC45A2	rs28777	C	A	NorthAsians	EastAsians	2.38E-04	5.38E-04	0.657895
SLC45A2	rs26722	C	T	NorthAsians	EastAsians	-4.67E-04	5.31E-04	0.378937
EXOC2	rs4959270	C	A	NorthAsians	EastAsians	-1.62E-04	5.30E-04	0.759375
TYRP1	rs1408799	T	C	NorthAsians	EastAsians	6.00E-06	6.46E-04	0.992184
TYRP1	rs683	A	C	NorthAsians	EastAsians	-0.001162	6.01E-04	0.053093

Biology Open • Supplementary information

BNC2	rs10756819	G	A	NorthAsians	EastAsians	3.40E-05	5.28E-04	0.948733
BNC2	rs12350739	G	A	NorthAsians	EastAsians	0.001035	6.65E-04	0.119833
DDB1	rs11230664	C	T	NorthAsians	EastAsians	0.002143	0.001209	0.076471
DDB1	rs7120594	T	C	NorthAsians	EastAsians	0.002143	0.001209	0.076471
DDB1	rs7948623	A	T	NorthAsians	EastAsians	1.30E-04	0.001274	0.918425
DDB1	rs1377457	C	A	NorthAsians	EastAsians	8.83E-04	0.001224	0.470813
TPCN2	rs35264875	A	T	NorthAsians	EastAsians	7.44E-04	6.52E-04	0.253889
TPCN2	rs3829241	G	A	NorthAsians	EastAsians	4.87E-04	5.30E-04	0.358337
TYR	rs1042602	C	A	NorthAsians	EastAsians	0.002093	7.99E-04	**0.008792**
TYR	rs1393350	G	A	NorthAsians	EastAsians	9.79E-04	9.13E-04	0.28368
TYR	rs1126809	G	A	NorthAsians	EastAsians	0.001632	0.001029	0.112903
KITLG	rs642742	A	G	NorthAsians	EastAsians	4.33E-04	5.40E-04	0.422821
KITLG	rs12821256	T	C	NorthAsians	EastAsians	7.83E-04	0.001359	0.564616
DCT	rs1407995	T	C	NorthAsians	EastAsians	1.19E-04	5.31E-04	0.822512
DCT	rs2031526	G	A	NorthAsians	EastAsians	-1.25E-04	5.31E-04	0.813192
SLC24A4	rs12896399	G	T	NorthAsians	EastAsians	-4.70E-05	5.30E-04	0.928955
SLC24A4	rs2402130	G	A	NorthAsians	EastAsians	-6.70E-04	5.43E-04	0.217046
OCA2	rs2311843	C	T	NorthAsians	EastAsians	-4.50E-05	5.33E-04	0.933321
OCA2	rs1800414	A	G	NorthAsians	EastAsians	-0.002186	5.52E-04	**7.40E-05**
OCA2	rs1800404	C	T	NorthAsians	EastAsians	0.0018	5.40E-04	**8.52E-04**
OCA2	rs1800401	C	T	NorthAsians	EastAsians	9.92E-04	6.41E-04	0.122014
HERC2	rs1129038	G	A	NorthAsians	EastAsians	0.001182	7.99E-04	0.139204
HERC2	rs12913832	A	G	NorthAsians	EastAsians	0.001375	8.22E-04	0.094386
HERC2	rs916977	A	G	NorthAsians	EastAsians	1.14E-04	5.30E-04	0.829383
HERC2	rs1667394	G	A	NorthAsians	EastAsians	1.16E-04	5.30E-04	0.826312
SLC24A5	rs1426654	G	A	NorthAsians	EastAsians	0.002017	5.61E-04	**3.21E-04**
MYO5A	rs4776053	C	T	NorthAsians	EastAsians	7.61E-04	5.30E-04	0.1509
MC1R	rs2228479	G	A	NorthAsians	EastAsians	2.90E-05	5.30E-04	0.956249
MC1R	rs885479	G	A	NorthAsians	EastAsians	5.40E-05	5.29E-04	0.918692

MFSD12	rs56203814	C	T	NorthAsians	EastAsians	0.001629	0.001624	0.315933
MFSD12	rs10424065	C	T	NorthAsians	EastAsians	7.83E-04	0.001359	0.564616
MFSD12	rs6510760	G	A	NorthAsians	EastAsians	-3.00E-06	5.71E-04	0.996409
MFSD12	rs112332856	T	C	NorthAsians	EastAsians	2.50E-04	8.55E-04	0.769913
ASIP	rs6119471	G	C	NorthAsians	EastAsians	-0.001629	0.001624	0.315933
ASIP	rs6058017	G	A	NorthAsians	EastAsians	0.002077	6.35E-04	**0.001071**
PIGU	rs2378249	A	G	NorthAsians	EastAsians	4.42E-04	5.32E-04	0.405868
SLC45A2	rs16891982	C	G	NorthAsians	EastAfricans	8.87E-04	7.11E-04	0.21234
SLC45A2	rs28777	C	A	NorthAsians	EastAfricans	-2.13E-04	6.41E-04	0.740327
SLC45A2	rs26722	C	T	NorthAsians	EastAfricans	6.67E-04	6.52E-04	0.306577
EXOC2	rs4959270	C	A	NorthAsians	EastAfricans	7.26E-04	6.52E-04	0.26576
TYRP1	rs1408799	T	C	NorthAsians	EastAfricans	-0.001054	6.63E-04	0.11218
TYRP1	rs683	A	C	NorthAsians	EastAfricans	6.40E-04	6.50E-04	0.325313
BNC2	rs10756819	G	A	NorthAsians	EastAfricans	0.001578	6.80E-04	**0.020383**
BNC2	rs12350739	G	A	NorthAsians	EastAfricans	6.41E-04	8.53E-04	0.45233
DDB1	rs11230664	C	T	NorthAsians	EastAfricans	0.003418	8.48E-04	**5.50E-05**
DDB1	rs7120594	T	C	NorthAsians	EastAfricans	0.0029	8.39E-04	**5.48E-04**
DDB1	rs7948623	A	T	NorthAsians	EastAfricans	-0.002597	8.38E-04	**0.001932**
DDB1	rs1377457	C	A	NorthAsians	EastAfricans	0.00348	8.50E-04	**4.20E-05**
TPCN2	rs35264875	A	T	NorthAsians	EastAfricans	-8.49E-04	6.62E-04	0.199751
TPCN2	rs3829241	G	A	NorthAsians	EastAfricans	0.001194	6.80E-04	0.079327
TYR	rs1042602	C	A	NorthAsians	EastAfricans	5.99E-04	8.55E-04	0.48367
TYR	rs1393350	G	A	NorthAsians	EastAfricans	1.71E-04	8.92E-04	0.848096
TYR	rs1126809	G	A	NorthAsians	EastAfricans	1.71E-04	8.92E-04	0.848096
KITLG	rs642742	A	G	NorthAsians	EastAfricans	0.001497	6.46E-04	**0.020482**
KITLG	rs12821256	T	C	NorthAsians	EastAfricans	-0.001347	8.46E-04	0.111491
DCT	rs1407995	T	C	NorthAsians	EastAfricans	-9.80E-04	6.42E-04	0.126771
DCT	rs2031526	G	A	NorthAsians	EastAfricans	0.001545	6.59E-04	**0.019011**
SLC24A4	rs12896399	G	T	NorthAsians	EastAfricans	7.73E-04	6.52E-04	0.235596

Biology Open • Supplementary information

SLC24A4	rs2402130	G	A	NorthAsians	EastAfricans	7.24E-04	6.40E-04	0.258123
OCA2	rs2311843	C	T	NorthAsians	EastAfricans	0.002135	7.09E-04	**0.002614**
OCA2	rs1800414	A	G	NorthAsians	EastAfricans	0.001157	8.40E-04	0.168583
OCA2	rs1800404	C	T	NorthAsians	EastAfricans	0.001969	6.63E-04	**0.002979**
OCA2	rs1800401	C	T	NorthAsians	EastAfricans	-1.46E-04	6.78E-04	0.829763
HERC2	rs1129038	G	A	NorthAsians	EastAfricans	3.69E-04	8.70E-04	0.671346
HERC2	rs12913832	A	G	NorthAsians	EastAfricans	3.69E-04	8.70E-04	0.671346
HERC2	rs916977	A	G	NorthAsians	EastAfricans	0.001139	6.81E-04	0.094325
HERC2	rs1667394	G	A	NorthAsians	EastAfricans	0.001139	6.81E-04	0.094325
SLC24A5	rs1426654	G	A	NorthAsians	EastAfricans	6.60E-04	6.70E-04	0.324444
MYO5A	rs4776053	C	T	NorthAsians	EastAfricans	8.34E-04	6.52E-04	0.200935
MC1R	rs2228479	G	A	NorthAsians	EastAfricans	0.001772	8.37E-04	**0.034127**
MC1R	rs885479	G	A	NorthAsians	EastAfricans	0.002309	8.36E-04	**0.005774**
MFSD12	rs56203814	C	T	NorthAsians	EastAfricans	-0.001695	8.40E-04	**0.043614**
MFSD12	rs10424065	C	T	NorthAsians	EastAfricans	-0.0025	8.37E-04	**0.002835**
MFSD12	rs6510760	G	A	NorthAsians	EastAfricans	-0.002355	6.68E-04	**4.27E-04**
MFSD12	rs112332856	T	C	NorthAsians	EastAfricans	-0.003174	7.30E-04	**1.40E-05**
ASIP	rs6119471	G	C	NorthAsians	EastAfricans	0.002038	8.38E-04	**0.014996**
ASIP	rs6058017	G	A	NorthAsians	EastAfricans	0.001952	6.63E-04	**0.003258**
PIGU	rs2378249	A	G	NorthAsians	EastAfricans	6.67E-04	6.52E-04	0.306577
SLC45A2	rs16891982	C	G	NorthAsians	WestAfricans	0.001087	5.44E-04	**0.045692**
SLC45A2	rs28777	C	A	NorthAsians	WestAfricans	2.00E-06	5.19E-04	0.997565
SLC45A2	rs26722	C	T	NorthAsians	WestAfricans	6.09E-04	5.20E-04	0.240886
EXOC2	rs4959270	C	A	NorthAsians	WestAfricans	1.29E-04	5.18E-04	0.803923
TYRP1	rs1408799	T	C	NorthAsians	WestAfricans	-7.09E-04	5.36E-04	0.185954
TYRP1	rs683	A	C	NorthAsians	WestAfricans	2.45E-04	5.26E-04	0.641416
BNC2	rs10756819	G	A	NorthAsians	WestAfricans	8.84E-04	5.19E-04	0.088369
BNC2	rs12350739	G	A	NorthAsians	WestAfricans	6.60E-04	5.67E-04	0.244361
DDB1	rs11230664	C	T	NorthAsians	WestAfricans	0.002097	6.71E-04	**0.001773**

Biology Open • Supplementary information

DDB1	rs7120594	T	C	NorthAsians	WestAfricans	0.00197	6.71E-04	**0.003326**
DDB1	rs7948623	A	T	NorthAsians	WestAfricans	-0.00149	6.71E-04	**0.026434**
DDB1	rs1377457	C	A	NorthAsians	WestAfricans	0.002116	6.71E-04	**0.001611**
TPCN2	rs35264875	A	T	NorthAsians	WestAfricans	3.46E-04	5.46E-04	0.525583
TPCN2	rs3829241	G	A	NorthAsians	WestAfricans	8.71E-04	5.21E-04	0.094207
TYR	rs1042602	C	A	NorthAsians	WestAfricans	5.24E-04	5.59E-04	0.348536
TYR	rs1393350	G	A	NorthAsians	WestAfricans	7.78E-04	7.15E-04	0.276486
TYR	rs1126809	G	A	NorthAsians	WestAfricans	1.87E-04	5.95E-04	0.752689
KITLG	rs642742	A	G	NorthAsians	WestAfricans	0.001333	5.20E-04	**0.010391**
KITLG	rs12821256	T	C	NorthAsians	WestAfricans	4.44E-04	7.96E-04	0.577185
DCT	rs1407995	T	C	NorthAsians	WestAfricans	-6.74E-04	5.18E-04	0.193307
DCT	rs2031526	G	A	NorthAsians	WestAfricans	0.001135	5.19E-04	**0.028743**
SLC24A4	rs12896399	G	T	NorthAsians	WestAfricans	0.001583	5.52E-04	**0.004124**
SLC24A4	rs2402130	G	A	NorthAsians	WestAfricans	7.00E-04	5.19E-04	0.177234
OCA2	rs2311843	C	T	NorthAsians	WestAfricans	0.001468	5.21E-04	**0.004867**
OCA2	rs1800414	A	G	NorthAsians	WestAfricans	0.001555	6.75E-04	**0.021184**
OCA2	rs1800404	C	T	NorthAsians	WestAfricans	0.001201	5.20E-04	**0.020896**
OCA2	rs1800401	C	T	NorthAsians	WestAfricans	-5.74E-04	5.31E-04	0.280179
HERC2	rs1129038	G	A	NorthAsians	WestAfricans	2.35E-04	5.67E-04	0.678681
HERC2	rs12913832	A	G	NorthAsians	WestAfricans	2.35E-04	5.67E-04	0.678681
HERC2	rs916977	A	G	NorthAsians	WestAfricans	4.95E-04	5.19E-04	0.33946
HERC2	rs1667394	G	A	NorthAsians	WestAfricans	5.38E-04	5.19E-04	0.30001
SLC24A5	rs1426654	G	A	NorthAsians	WestAfricans	3.62E-04	5.21E-04	0.486252
MYO5A	rs4776053	C	T	NorthAsians	WestAfricans	5.67E-04	5.18E-04	0.273699
MC1R	rs2228479	G	A	NorthAsians	WestAfricans	0.00204	6.72E-04	**0.002397**
MC1R	rs885479	G	A	NorthAsians	WestAfricans	0.002462	6.72E-04	**2.46E-04**
MFSD12	rs56203814	C	T	NorthAsians	WestAfricans	-0.001463	6.71E-04	**0.029258**
MFSD12	rs10424065	C	T	NorthAsians	WestAfricans	-0.001619	6.71E-04	**0.01582**
MFSD12	rs6510760	G	A	NorthAsians	WestAfricans	-0.001475	5.24E-04	**0.004891**

Biology Open • Supplementary information

MFSD12	rs112332856	T	C	NorthAsians	WestAfricans	-0.001961	5.73E-04	**6.16E-04**
ASIP	rs6119471	G	C	NorthAsians	WestAfricans	0.002184	6.71E-04	**0.001134**
ASIP	rs6058017	G	A	NorthAsians	WestAfricans	0.001673	5.36E-04	**0.001802**
PIGU	rs2378249	A	G	NorthAsians	WestAfricans	1.95E-04	5.18E-04	0.707335
SLC45A2	rs16891982	C	G	Oceanians	EastAsians	0.001089	6.67E-04	0.102701
SLC45A2	rs28777	C	A	Oceanians	EastAsians	8.66E-04	6.28E-04	0.167747
SLC45A2	rs26722	C	T	Oceanians	EastAsians	-3.81E-04	6.32E-04	0.546907
EXOC2	rs4959270	C	A	Oceanians	EastAsians	0.001306	6.43E-04	**0.042308**
TYRP1	rs1408799	T	C	Oceanians	EastAsians	8.90E-05	7.45E-04	0.904817
TYRP1	rs683	A	C	Oceanians	EastAsians	-4.27E-04	7.49E-04	0.568553
BNC2	rs10756819	G	A	Oceanians	EastAsians	-1.10E-04	6.27E-04	0.860449
BNC2	rs12350739	G	A	Oceanians	EastAsians	1.45E-04	9.51E-04	0.8785
DDB1	rs11230664	C	T	Oceanians	EastAsians	-0.001606	6.30E-04	**0.010798**
DDB1	rs7120594	T	C	Oceanians	EastAsians	-0.001606	6.30E-04	**0.010798**
DDB1	rs7948623	A	T	Oceanians	EastAsians	0.002989	6.82E-04	**1.20E-05**
DDB1	rs1377457	C	A	Oceanians	EastAsians	-0.002425	6.42E-04	**1.59E-04**
TPCN2	rs35264875	A	T	Oceanians	EastAsians	0.002225	6.40E-04	**5.03E-04**
TPCN2	rs3829241	G	A	Oceanians	EastAsians	-7.46E-04	6.73E-04	0.267951
TYR	rs1042602	C	A	Oceanians	EastAsians	8.89E-04	9.91E-04	0.369733
TYR	rs1393350	G	A	Oceanians	EastAsians	7.20E-04	9.76E-04	0.460701
TYR	rs1126809	G	A	Oceanians	EastAsians	0.001144	0.001024	0.26368
KITLG	rs642742	A	G	Oceanians	EastAsians	2.10E-05	6.34E-04	0.973094
KITLG	rs12821256	T	C	Oceanians	EastAsians	0.001144	0.001024	0.26368
DCT	rs1407995	T	C	Oceanians	EastAsians	6.52E-04	6.28E-04	0.298811
DCT	rs2031526	G	A	Oceanians	EastAsians	-6.56E-04	6.28E-04	0.295756
SLC24A4	rs12896399	G	T	Oceanians	EastAsians	5.03E-04	6.28E-04	0.422541
SLC24A4	rs2402130	G	A	Oceanians	EastAsians	-5.17E-04	6.40E-04	0.418887
OCA2	rs2311843	C	T	Oceanians	EastAsians	-5.32E-04	6.28E-04	0.396381
OCA2	rs1800414	A	G	Oceanians	EastAsians	-8.29E-04	6.33E-04	0.190345

Biology Open • Supplementary information

OCA2	rs1800404	C	T	Oceanians	EastAsians	-1.68E-04	6.29E-04	0.789951
OCA2	rs1800401	C	T	Oceanians	EastAsians	2.40E-05	9.48E-04	0.979833
HERC2	rs1129038	G	A	Oceanians	EastAsians	5.94E-04	9.68E-04	0.539336
HERC2	rs12913832	A	G	Oceanians	EastAsians	7.20E-04	9.76E-04	0.460701
HERC2	rs916977	A	G	Oceanians	EastAsians	4.50E-04	6.27E-04	0.473124
HERC2	rs1667394	G	A	Oceanians	EastAsians	4.51E-04	6.27E-04	0.471789
SLC24A5	rs1426654	G	A	Oceanians	EastAsians	3.31E-04	7.48E-04	0.658389
MYO5A	rs4776053	C	T	Oceanians	EastAsians	-0.001076	7.41E-04	0.146351
MC1R	rs2228479	G	A	Oceanians	EastAsians	-6.02E-04	6.47E-04	0.351911
MC1R	rs885479	G	A	Oceanians	EastAsians	-0.00184	6.94E-04	**0.008036**
MFSD12	rs56203814	C	T	Oceanians	EastAsians	0.001694	0.001175	0.149534
MFSD12	rs10424065	C	T	Oceanians	EastAsians	0.001144	0.001024	0.26368
MFSD12	rs6510760	G	A	Oceanians	EastAsians	0.001475	6.29E-04	**0.019026**
MFSD12	rs112332856	T	C	Oceanians	EastAsians	0.002203	6.52E-04	**7.21E-04**
ASIP	rs6119471	G	C	Oceanians	EastAsians	-0.001694	0.001175	0.149534
ASIP	rs6058017	G	A	Oceanians	EastAsians	2.40E-04	6.38E-04	0.707433
PIGU	rs2378249	A	G	Oceanians	EastAsians	-1.03E-04	6.41E-04	0.872447
SLC45A2	rs16891982	C	G	Oceanians	EastAfricans	7.26E-04	7.29E-04	0.318758
SLC45A2	rs28777	C	A	Oceanians	EastAfricans	3.35E-04	6.44E-04	0.603448
SLC45A2	rs26722	C	T	Oceanians	EastAfricans	6.08E-04	6.59E-04	0.356442
EXOC2	rs4959270	C	A	Oceanians	EastAfricans	0.001811	6.65E-04	**0.006465**
TYRP1	rs1408799	T	C	Oceanians	EastAfricans	-9.88E-04	7.12E-04	0.164947
TYRP1	rs683	A	C	Oceanians	EastAfricans	8.92E-04	7.13E-04	0.210637
BNC2	rs10756819	G	A	Oceanians	EastAfricans	0.001476	6.84E-04	**0.031069**
BNC2	rs12350739	G	A	Oceanians	EastAfricans	2.35E-04	9.97E-04	0.813429
DDB1	rs11230664	C	T	Oceanians	EastAfricans	0.001111	6.56E-04	0.090412
DDB1	rs7120594	T	C	Oceanians	EastAfricans	5.94E-04	6.45E-04	0.357396
DDB1	rs7948623	A	T	Oceanians	EastAfricans	-3.64E-04	6.43E-04	0.57195
DDB1	rs1377457	C	A	Oceanians	EastAfricans	0.001174	6.59E-04	0.074815

Biology Open • Supplementary information

TPCN2	rs35264875	A	T	Oceanians	EastAfricans	4.90E-04	6.46E-04	0.448032
TPCN2	rs3829241	G	A	Oceanians	EastAfricans	3.77E-04	7.10E-04	0.59576
TYR	rs1042602	C	A	Oceanians	EastAfricans	2.35E-04	9.97E-04	0.813429
TYR	rs1393350	G	A	Oceanians	EastAfricans	2.35E-04	9.97E-04	0.813429
TYR	rs1126809	G	A	Oceanians	EastAfricans	2.35E-04	9.97E-04	0.813429
KITLG	rs642742	A	G	Oceanians	EastAfricans	0.001297	6.52E-04	**0.04667**
KITLG	rs12821256	T	C	Oceanians	EastAfricans	-8.58E-04	8.47E-04	0.310831
DCT	rs1407995	T	C	Oceanians	EastAfricans	-5.38E-04	6.46E-04	0.404862
DCT	rs2031526	G	A	Oceanians	EastAfricans	0.001103	6.63E-04	0.096146
SLC24A4	rs12896399	G	T	Oceanians	EastAfricans	0.001184	6.56E-04	0.071209
SLC24A4	rs2402130	G	A	Oceanians	EastAfricans	6.62E-04	6.49E-04	0.308068
OCA2	rs2311843	C	T	Oceanians	EastAfricans	0.001748	7.13E-04	**0.014191**
OCA2	rs1800414	A	G	Oceanians	EastAfricans	0.001612	8.42E-04	0.055664
OCA2	rs1800404	C	T	Oceanians	EastAfricans	9.40E-04	6.66E-04	0.158276
OCA2	rs1800401	C	T	Oceanians	EastAfricans	-6.23E-04	8.55E-04	0.466227
HERC2	rs1129038	G	A	Oceanians	EastAfricans	2.35E-04	9.97E-04	0.813429
HERC2	rs12913832	A	G	Oceanians	EastAfricans	2.35E-04	9.97E-04	0.813429
HERC2	rs916977	A	G	Oceanians	EastAfricans	0.001428	6.85E-04	**0.036998**
HERC2	rs1667394	G	A	Oceanians	EastAfricans	0.001428	6.85E-04	**0.036998**
SLC24A5	rs1426654	G	A	Oceanians	EastAfricans	-9.40E-05	7.37E-04	0.898072
MYO5A	rs4776053	C	T	Oceanians	EastAfricans	-3.75E-04	7.23E-04	0.604181
MC1R	rs2228479	G	A	Oceanians	EastAfricans	0.001295	8.48E-04	0.126915
MC1R	rs885479	G	A	Oceanians	EastAfricans	8.67E-04	8.70E-04	0.319434
MFSD12	rs56203814	C	T	Oceanians	EastAfricans	-0.001207	8.41E-04	0.151188
MFSD12	rs10424065	C	T	Oceanians	EastAfricans	-0.002011	8.38E-04	**0.016387**
MFSD12	rs6510760	G	A	Oceanians	EastAfricans	-0.001219	6.65E-04	0.066782
MFSD12	rs112332856	T	C	Oceanians	EastAfricans	-0.001604	6.67E-04	**0.016127**
ASIP	rs6119471	G	C	Oceanians	EastAfricans	0.00155	8.38E-04	0.06457
ASIP	rs6058017	G	A	Oceanians	EastAfricans	0.001098	6.51E-04	0.091568

Biology Open • Supplementary information

PIGU	rs2378249	A	G	Oceanians	EastAfricans	3.67E-04	6.64E-04	0.580531
SLC45A2	rs16891982	C	G	Oceanians	WestAfricans	9.61E-04	5.60E-04	0.086576
SLC45A2	rs28777	C	A	Oceanians	WestAfricans	4.33E-04	5.24E-04	0.408818
SLC45A2	rs26722	C	T	Oceanians	WestAfricans	5.63E-04	5.27E-04	0.286
EXOC2	rs4959270	C	A	Oceanians	WestAfricans	9.84E-04	5.31E-04	0.063704
TYRP1	rs1408799	T	C	Oceanians	WestAfricans	-6.57E-04	5.76E-04	0.253421
TYRP1	rs683	A	C	Oceanians	WestAfricans	4.44E-04	5.76E-04	0.441185
BNC2	rs10756819	G	A	Oceanians	WestAfricans	8.03E-04	5.25E-04	0.125638
BNC2	rs12350739	G	A	Oceanians	WestAfricans	3.40E-04	7.00E-04	0.626702
DDB1	rs11230664	C	T	Oceanians	WestAfricans	2.80E-04	5.24E-04	0.592903
DDB1	rs7120594	T	C	Oceanians	WestAfricans	1.52E-04	5.23E-04	0.771004
DDB1	rs7948623	A	T	Oceanians	WestAfricans	2.70E-04	5.24E-04	0.605794
DDB1	rs1377457	C	A	Oceanians	WestAfricans	2.99E-04	5.24E-04	0.568196
TPCN2	rs35264875	A	T	Oceanians	WestAfricans	0.001402	5.36E-04	**0.008884**
TPCN2	rs3829241	G	A	Oceanians	WestAfricans	2.27E-04	5.47E-04	0.677517
TYR	rs1042602	C	A	Oceanians	WestAfricans	2.38E-04	6.92E-04	0.730883
TYR	rs1393350	G	A	Oceanians	WestAfricans	8.29E-04	7.98E-04	0.299012
TYR	rs1126809	G	A	Oceanians	WestAfricans	2.38E-04	6.92E-04	0.730883
KITLG	rs642742	A	G	Oceanians	WestAfricans	0.001175	5.27E-04	**0.025836**
KITLG	rs12821256	T	C	Oceanians	WestAfricans	8.29E-04	7.98E-04	0.299012
DCT	rs1407995	T	C	Oceanians	WestAfricans	-3.25E-04	5.24E-04	0.534328
DCT	rs2031526	G	A	Oceanians	WestAfricans	7.87E-04	5.25E-04	0.133799
SLC24A4	rs12896399	G	T	Oceanians	WestAfricans	0.001906	5.57E-04	**6.26E-04**
SLC24A4	rs2402130	G	A	Oceanians	WestAfricans	6.51E-04	5.28E-04	0.217798
OCA2	rs2311843	C	T	Oceanians	WestAfricans	0.001163	5.27E-04	**0.027279**
OCA2	rs1800414	A	G	Oceanians	WestAfricans	0.001913	6.78E-04	**0.004789**
OCA2	rs1800404	C	T	Oceanians	WestAfricans	3.90E-04	5.25E-04	0.457676
OCA2	rs1800401	C	T	Oceanians	WestAfricans	-9.50E-04	6.74E-04	0.158555
HERC2	rs1129038	G	A	Oceanians	WestAfricans	1.29E-04	6.87E-04	0.850487

Biology Open • Supplementary information

HERC2	rs12913832	A	G	Oceanians	WestAfricans	1.29E-04	6.87E-04	0.850487
HERC2	rs916977	A	G	Oceanians	WestAfricans	7.23E-04	5.24E-04	0.167892
HERC2	rs1667394	G	A	Oceanians	WestAfricans	7.65E-04	5.25E-04	0.144519
SLC24A5	rs1426654	G	A	Oceanians	WestAfricans	-2.32E-04	5.77E-04	0.687656
MYO5A	rs4776053	C	T	Oceanians	WestAfricans	-3.85E-04	5.76E-04	0.504393
MC1R	rs2228479	G	A	Oceanians	WestAfricans	0.001664	6.83E-04	**0.014853**
MC1R	rs885479	G	A	Oceanians	WestAfricans	0.001326	7.00E-04	0.05814
MFSD12	rs56203814	C	T	Oceanians	WestAfricans	-0.001078	6.73E-04	0.109476
MFSD12	rs10424065	C	T	Oceanians	WestAfricans	-0.001234	6.73E-04	0.066848
MFSD12	rs6510760	G	A	Oceanians	WestAfricans	-5.80E-04	5.24E-04	0.268324
MFSD12	rs112332856	T	C	Oceanians	WestAfricans	-7.24E-04	5.25E-04	0.167624
ASIP	rs6119471	G	C	Oceanians	WestAfricans	0.001799	6.73E-04	**0.007547**
ASIP	rs6058017	G	A	Oceanians	WestAfricans	9.99E-04	5.29E-04	0.058665
PIGU	rs2378249	A	G	Oceanians	WestAfricans	-4.20E-05	5.30E-04	0.936817
SLC45A2	rs16891982	C	G	EastAsians	EastAfricans	-1.11E-04	7.02E-04	0.873917
SLC45A2	rs28777	C	A	EastAsians	EastAfricans	-3.32E-04	6.27E-04	0.596567
SLC45A2	rs26722	C	T	EastAsians	EastAfricans	9.01E-04	6.39E-04	0.158605
EXOC2	rs4959270	C	A	EastAsians	EastAfricans	8.07E-04	6.39E-04	0.206507
TYRP1	rs1408799	T	C	EastAsians	EastAfricans	-0.001057	6.29E-04	0.093125
TYRP1	rs683	A	C	EastAsians	EastAfricans	0.001221	6.33E-04	0.053946
BNC2	rs10756819	G	A	EastAsians	EastAfricans	0.001561	6.68E-04	**0.019443**
BNC2	rs12350739	G	A	EastAsians	EastAfricans	1.24E-04	8.34E-04	0.882304
DDB1	rs11230664	C	T	EastAsians	EastAfricans	0.002346	6.40E-04	**2.48E-04**
DDB1	rs7120594	T	C	EastAsians	EastAfricans	0.001829	6.29E-04	**0.003637**
DDB1	rs7948623	A	T	EastAsians	EastAfricans	-0.002662	6.58E-04	**5.20E-05**
DDB1	rs1377457	C	A	EastAsians	EastAfricans	0.003039	6.50E-04	**3.00E-06**
TPCN2	rs35264875	A	T	EastAsians	EastAfricans	-0.001222	6.35E-04	0.05446
TPCN2	rs3829241	G	A	EastAsians	EastAfricans	9.51E-04	6.68E-04	0.154736
TYR	rs1042602	C	A	EastAsians	EastAfricans	-4.48E-04	8.61E-04	0.602762

TYR	rs1393350	G	A	EastAsians	EastAfricans	-3.19E-04	8.51E-04	0.708248
TYR	rs1126809	G	A	EastAsians	EastAfricans	-6.45E-04	8.84E-04	0.465599
KITLG	rs642742	A	G	EastAsians	EastAfricans	0.001281	6.31E-04	**0.0423**
KITLG	rs12821256	T	C	EastAsians	EastAfricans	-0.001738	7.10E-04	**0.014326**
DCT	rs1407995	T	C	EastAsians	EastAfricans	-0.00104	6.28E-04	0.098027
DCT	rs2031526	G	A	EastAsians	EastAfricans	0.001607	6.45E-04	**0.012763**
SLC24A4	rs12896399	G	T	EastAsians	EastAfricans	7.97E-04	6.39E-04	0.212191
SLC24A4	rs2402130	G	A	EastAsians	EastAfricans	0.001059	6.26E-04	0.090478
OCA2	rs2311843	C	T	EastAsians	EastAfricans	0.002157	6.97E-04	**0.001962**
OCA2	rs1800414	A	G	EastAsians	EastAfricans	0.002249	8.26E-04	**0.006478**
OCA2	rs1800404	C	T	EastAsians	EastAfricans	0.001069	6.48E-04	0.099004
OCA2	rs1800401	C	T	EastAsians	EastAfricans	-6.41E-04	6.56E-04	0.327988
HERC2	rs1129038	G	A	EastAsians	EastAfricans	-2.22E-04	8.46E-04	0.79322
HERC2	rs12913832	A	G	EastAsians	EastAfricans	-3.19E-04	8.51E-04	0.708248
HERC2	rs916977	A	G	EastAsians	EastAfricans	0.001082	6.68E-04	0.105386
HERC2	rs1667394	G	A	EastAsians	EastAfricans	0.00108	6.68E-04	0.105724
SLC24A5	rs1426654	G	A	EastAsians	EastAfricans	-3.49E-04	6.60E-04	0.597169
MYO5A	rs4776053	C	T	EastAsians	EastAfricans	4.53E-04	6.39E-04	0.478246
MC1R	rs2228479	G	A	EastAsians	EastAfricans	0.001758	8.26E-04	**0.033387**
MC1R	rs885479	G	A	EastAsians	EastAfricans	0.002282	8.26E-04	**0.005752**
MFSD12	rs56203814	C	T	EastAsians	EastAfricans	-0.00251	8.31E-04	**0.002534**
MFSD12	rs10424065	C	T	EastAsians	EastAfricans	-0.002891	6.99E-04	**3.60E-05**
MFSD12	rs6510760	G	A	EastAsians	EastAfricans	-0.002354	6.49E-04	**2.86E-04**
MFSD12	rs112332856	T	C	EastAsians	EastAfricans	-0.003299	6.60E-04	**1.00E-06**
ASIP	rs6119471	G	C	EastAsians	EastAfricans	0.002852	8.29E-04	**5.79E-04**
ASIP	rs6058017	G	A	EastAsians	EastAfricans	9.13E-04	6.27E-04	0.144925
PIGU	rs2378249	A	G	EastAsians	EastAfricans	4.46E-04	6.39E-04	0.485309
SLC45A2	rs16891982	C	G	EastAsians	WestAfricans	3.00E-04	5.36E-04	0.575049
SLC45A2	rs28777	C	A	EastAsians	WestAfricans	-9.20E-05	5.07E-04	0.85544

SLC45A2	rs26722	C	T	EastAsians	WestAfricans	7.93E-04	5.08E-04	0.118286
EXOC2	rs4959270	C	A	EastAsians	WestAfricans	1.93E-04	5.06E-04	0.703733
TYRP1	rs1408799	T	C	EastAsians	WestAfricans	-7.11E-04	5.09E-04	0.16208
TYRP1	rs683	A	C	EastAsians	WestAfricans	7.02E-04	5.11E-04	0.169651
BNC2	rs10756819	G	A	EastAsians	WestAfricans	8.70E-04	5.07E-04	0.086276
BNC2	rs12350739	G	A	EastAsians	WestAfricans	2.52E-04	5.48E-04	0.64552
DDB1	rs11230664	C	T	EastAsians	WestAfricans	0.001253	5.07E-04	**0.013528**
DDB1	rs7120594	T	C	EastAsians	WestAfricans	0.001126	5.07E-04	**0.026524**
DDB1	rs7948623	A	T	EastAsians	WestAfricans	-0.001541	5.31E-04	**0.003738**
DDB1	rs1377457	C	A	EastAsians	WestAfricans	0.001768	5.13E-04	**5.67E-04**
TPCN2	rs35264875	A	T	EastAsians	WestAfricans	5.30E-05	5.24E-04	0.919283
TPCN2	rs3829241	G	A	EastAsians	WestAfricans	6.80E-04	5.09E-04	0.182182
TYR	rs1042602	C	A	EastAsians	WestAfricans	-3.00E-04	5.65E-04	0.59469
TYR	rs1393350	G	A	EastAsians	WestAfricans	3.92E-04	6.82E-04	0.565332
TYR	rs1126809	G	A	EastAsians	WestAfricans	-4.55E-04	5.86E-04	0.437031
KITLG	rs642742	A	G	EastAsians	WestAfricans	0.001162	5.07E-04	**0.021928**
KITLG	rs12821256	T	C	EastAsians	WestAfricans	1.35E-04	7.08E-04	0.8484
DCT	rs1407995	T	C	EastAsians	WestAfricans	-7.21E-04	5.06E-04	0.154743
DCT	rs2031526	G	A	EastAsians	WestAfricans	0.001185	5.08E-04	**0.019612**
SLC24A4	rs12896399	G	T	EastAsians	WestAfricans	0.001601	5.41E-04	**0.00308**
SLC24A4	rs2402130	G	A	EastAsians	WestAfricans	9.65E-04	5.07E-04	0.057024
OCA2	rs2311843	C	T	EastAsians	WestAfricans	0.001485	5.10E-04	**0.00356**
OCA2	rs1800414	A	G	EastAsians	WestAfricans	0.002416	6.63E-04	**2.69E-04**
OCA2	rs1800404	C	T	EastAsians	WestAfricans	4.91E-04	5.07E-04	0.332155
OCA2	rs1800401	C	T	EastAsians	WestAfricans	-9.64E-04	5.13E-04	0.06006
HERC2	rs1129038	G	A	EastAsians	WestAfricans	-2.31E-04	5.43E-04	0.670853
HERC2	rs12913832	A	G	EastAsians	WestAfricans	-3.07E-04	5.48E-04	0.575425
HERC2	rs916977	A	G	EastAsians	WestAfricans	4.50E-04	5.07E-04	0.374526
HERC2	rs1667394	G	A	EastAsians	WestAfricans	4.92E-04	5.07E-04	0.332331

SLC24A5	rs1426654	G	A	EastAsians	WestAfricans	-4.32E-04	5.12E-04	0.398198
MYO5A	rs4776053	C	T	EastAsians	WestAfricans	2.68E-04	5.07E-04	0.597823
MC1R	rs2228479	G	A	EastAsians	WestAfricans	0.002028	6.63E-04	**0.00222**
MC1R	rs885479	G	A	EastAsians	WestAfricans	0.002441	6.63E-04	**2.32E-04**
MFSD12	rs56203814	C	T	EastAsians	WestAfricans	-0.002104	6.63E-04	**0.001509**
MFSD12	rs10424065	C	T	EastAsians	WestAfricans	-0.001928	5.63E-04	**6.22E-04**
MFSD12	rs6510760	G	A	EastAsians	WestAfricans	-0.001474	5.07E-04	**0.003679**
MFSD12	rs112332856	T	C	EastAsians	WestAfricans	-0.00206	5.16E-04	**6.60E-05**
ASIP	rs6119471	G	C	EastAsians	WestAfricans	0.002826	6.63E-04	**2.00E-05**
ASIP	rs6058017	G	A	EastAsians	WestAfricans	8.54E-04	5.07E-04	0.091702
PIGU	rs2378249	A	G	EastAsians	WestAfricans	2.00E-05	5.07E-04	0.967942
SLC45A2	rs16891982	C	G	EastAfricans	WestAfricans	3.88E-04	3.41E-04	0.255086
SLC45A2	rs28777	C	A	EastAfricans	WestAfricans	1.69E-04	1.81E-04	0.351565
SLC45A2	rs26722	C	T	EastAfricans	WestAfricans	8.40E-05	2.10E-04	0.689776
EXOC2	rs4959270	C	A	EastAfricans	WestAfricans	-4.43E-04	2.06E-04	**0.03162**
TYRP1	rs1408799	T	C	EastAfricans	WestAfricans	1.22E-04	1.82E-04	0.503424
TYRP1	rs683	A	C	EastAfricans	WestAfricans	-2.59E-04	1.85E-04	0.160278
BNC2	rs10756819	G	A	EastAfricans	WestAfricans	-3.59E-04	2.59E-04	0.165122
BNC2	rs12350739	G	A	EastAfricans	WestAfricans	1.55E-04	4.99E-04	0.756223
DDB1	rs11230664	C	T	EastAfricans	WestAfricans	-5.95E-04	2.06E-04	**0.003833**
DDB1	rs7120594	T	C	EastAfricans	WestAfricans	-3.15E-04	1.83E-04	0.084296
DDB1	rs7948623	A	T	EastAfricans	WestAfricans	5.57E-04	1.79E-04	**0.001852**
DDB1	rs1377457	C	A	EastAfricans	WestAfricans	-6.26E-04	2.11E-04	**0.003034**
TPCN2	rs35264875	A	T	EastAfricans	WestAfricans	0.001016	2.16E-04	**3.00E-06**
TPCN2	rs3829241	G	A	EastAfricans	WestAfricans	-6.90E-05	2.63E-04	0.792385
TYR	rs1042602	C	A	EastAfricans	WestAfricans	5.30E-05	4.89E-04	0.914067
TYR	rs1393350	G	A	EastAfricans	WestAfricans	6.43E-04	6.29E-04	0.306763
TYR	rs1126809	G	A	EastAfricans	WestAfricans	5.30E-05	4.89E-04	0.914067
KITLG	rs642742	A	G	EastAfricans	WestAfricans	1.53E-04	1.92E-04	0.423979

Biology Open • Supplementary information

KITLG	rs12821256	T	C	EastAfricans	WestAfricans	0.001505	4.73E-04	**0.001479**
DCT	rs1407995	T	C	EastAfricans	WestAfricans	9.90E-05	1.85E-04	0.594727
DCT	rs2031526	G	A	EastAfricans	WestAfricans	-8.20E-05	2.21E-04	0.712095
SLC24A4	rs12896399	G	T	EastAfricans	WestAfricans	9.73E-04	2.81E-04	**5.26E-04**
SLC24A4	rs2402130	G	A	EastAfricans	WestAfricans	1.30E-04	1.78E-04	0.464636
OCA2	rs2311843	C	T	EastAfricans	WestAfricans	-2.14E-04	3.06E-04	0.483851
OCA2	rs1800414	A	G	EastAfricans	WestAfricans	6.43E-04	6.29E-04	0.306763
OCA2	rs1800404	C	T	EastAfricans	WestAfricans	-3.51E-04	2.24E-04	0.11748
OCA2	rs1800401	C	T	EastAfricans	WestAfricans	-4.59E-04	2.24E-04	**0.040104**
HERC2	rs1129038	G	A	EastAfricans	WestAfricans	-5.60E-05	4.81E-04	0.90728
HERC2	rs12913832	A	G	EastAfricans	WestAfricans	-5.60E-05	4.81E-04	0.90728
HERC2	rs916977	A	G	EastAfricans	WestAfricans	-4.02E-04	2.59E-04	0.120361
HERC2	rs1667394	G	A	EastAfricans	WestAfricans	-3.59E-04	2.59E-04	0.165122
SLC24A5	rs1426654	G	A	EastAfricans	WestAfricans	-1.57E-04	2.39E-04	0.509837
MYO5A	rs4776053	C	T	EastAfricans	WestAfricans	-8.90E-05	2.08E-04	0.66729
MC1R	rs2228479	G	A	EastAfricans	WestAfricans	6.43E-04	6.29E-04	0.306763
MC1R	rs885479	G	A	EastAfricans	WestAfricans	6.43E-04	6.29E-04	0.306763
MFSD12	rs56203814	C	T	EastAfricans	WestAfricans	-1.27E-04	1.86E-04	0.494038
MFSD12	rs10424065	C	T	EastAfricans	WestAfricans	3.51E-04	1.78E-04	**0.048901**
MFSD12	rs6510760	G	A	EastAfricans	WestAfricans	3.81E-04	2.24E-04	0.089162
MFSD12	rs112332856	T	C	EastAfricans	WestAfricans	5.39E-04	2.23E-04	**0.015703**
ASIP	rs6119471	G	C	EastAfricans	WestAfricans	5.78E-04	1.79E-04	**0.001269**
ASIP	rs6058017	G	A	EastAfricans	WestAfricans	1.35E-04	1.82E-04	0.458646
PIGU	rs2378249	A	G	EastAfricans	WestAfricans	-3.31E-04	2.07E-04	0.109172

Biology Open • Supplementary information
